# Computational study of SENP1 in cancer by novel natural compounds and ZINC database screening

**DOI:** 10.3389/fphar.2023.1144632

**Published:** 2023-07-12

**Authors:** Somayye Taghvaei, Alireza Taghvaei, Mohammad Saberi Anvar, Chun Guo, Farzaneh Sabouni, Zarrin Minuchehr

**Affiliations:** ^1^ Department of Medical Biotechnology, National Institute of Genetic Engineering and Biotechnology, Tehran, Iran; ^2^ Faculty of Pharmacy, Hamedan University of Medical Sciences, Hamedan, Iran; ^3^ Department of Systems Biotechnology, National Institute of Genetic Engineering and Biotechnology, Tehran, Iran; ^4^ School of Biosciences, University of Sheffield, Sheffield, United Kingdom

**Keywords:** SENP1, natural compounds, ZINC database, resveratrol, cancer, molecular docking, molecular dynamics simulation

## Abstract

**Introduction:** Sentrin-specific protease 1 (SENP1) is a protein whose main function is deSUMOylation. SENP1 inhibits apoptosis, and increases angiogenesis, estrogen and androgen receptor transcription and c-Jun transcription factor, proliferation, growth, cell migration, and invasion of cancer. The *in vivo* and *in vitro* studies also demonstrated which natural compounds, especially phytochemicals, minerals, and vitamins, prevent cancer. More than 3,000 plant species have been reported in modern medicine. Natural compounds have many anti-cancerous andanti-turmeric properties such as antioxidative, antiangiogenic, antiproliferative, and pro-apoptotic properties.

**Methods:** In this study, we investigated the interaction of some natural compounds with SENP1 to inhibit its activity. We also screened the ZINC database including natural compounds. Molecular docking was performed, and toxicity of compounds was determined; then, molecular dynamics simulation (MDS) and essential dynamics (ED) were performed on natural compounds with higher free binding energies and minimal side effects. By searching in a large library, virtual screening of the ZINC database was performed using LibDock and CDOCKER, and the final top 20 compounds were allowed for docking against SENP1. According to the docking study, the top three leading molecules were selected and further analyzed by MDS and ED.

**Results:** The results suggest that resveratrol (from the selected compounds) and ZINC33916875 (from the ZINC database) could be more promising SENP1 inhibitory ligands.

**Discussion:** Because these compounds can inhibit SENP1 activity, then they can be novel candidates for cancer treatment. However, wet laboratory experiments are needed to validate their efficacy as SENP1 inhibitors.

## 1 Introduction

Cancer is one of the leading causes of mortality worldwide. Chemotherapy and radiotherapy have been reported to be considerable in cancer therapy, but these treatments have side effects. Then, anticancer drugs based on medicinal plants have been paid attention to because of their lowest side effects. The beneficial effects of herbal medications were shown on immunological regulation, survival, and quality of life in cancer patients, alone or using a combination of herbal medications with diverse mechanisms in clinical trial-based studies (Almatroodi et al., 2022).

Sentrin-specific protease 1 (SENP1) is located at 12q13.11 in humans. SENP1 prefers small ubiquitin-like modifier (SUMO1) over SUMO2/3 for deSUMOylation (Kim and Baek, 2009). Decreased expression of SENP1 by RNA interference increased total SUMOylated proteins and progressive multifocal leukoencephalopathy (PML) counts plus P53-mediated transcriptional activity and premature aging (Andreou and Tavernarakis, 2010a). Deregulation in the SUMO pathway contributes to oncogenic transformation by affecting the SUMOylation/deSUMOylation of many oncoproteins and tumor suppressors. A loss of balance between SUMOylation and deSUMOylation has been reported in several studies and in various disease types including cancer (Taghvaei et al., 2021a). SENP1 enhances the transcriptional activity of androgen receptor (AR), eases c-Jun-dependent transcription, and induces expression of the cell cycle regulator (Cyclin D1) (Taghvaei et al., 2021a). SENP1 deletion has prevented cell growth by the upregulation of CDK inhibitors, such as p21 and p16 *in vitro* and *in vivo* growth of colon cancer cells (Xu et al., 2011). Prostate cancer cell growth could be induced because hypoxia-inducible factor 1 alpha (HIF-1α) activation and stabilization by SENP1 result in promoted cyclin D1 and vascular endothelial growth factor (VEGF) levels, angiogenesis, and cell growth (Cheng et al., 2006). SENP1 regulates *matrix metalloproteinase 2* (*MMP2*) and *MMP9* expression. This introduces SENP1 in the progression of prostate cancer and suggests SENP1 as a prognostic marker and a therapeutic target for prostate cancer metastasis patients (Wang Q. et al., 2013). Moreover, SENP1 was upregulated in pancreatic ductal adenocarcinoma (PDAC) tissues compared with adjacent normal tissues. Silencing of SENP1 led to *MMP-9* downregulation, which is fundamental for PDAC cell growth and migration (Ma et al., 2014).

The deSUMOylation of P300 induces c-Jun activity and increases cyclin D expression. SENP1 might be utilized as a molecular target for the discovery of antitumor drugs *vs.* human hepatocellular carcinoma (HCC) metastasis. Data represented SENP1 knockdown leads to the inhibition of HGF-induced proliferation and migration at the same time (Taghvaei et al., 2021b). SENP1 was reported to be involved in hepatocarcinogenesis through the regulation of HIF-1α deSUMOylation in hypoxia conditions. Novel inhibitor development that particularly targets SENP1 may offer a new therapeutic approach to block development, metastasis, and recurrence of HCC (Cui et al., 2017). An increased expression of SENP1 has also been reported in thyroid adenomas (Jacques et al., 2005). SENP1 can also cause glioma, multiple myeloma, and lung, breast, and bladder cancers (Brems-Eskildsen et al., 2010; Wang et al., 2013; Xu et al., 2015; Wang et al., 2016; Xiang‐ming et al., 2016; Zhang et al., 2016).

SENP1 is located in PML bodies and can process all SUMO1-3 precursors. In mitotic cells, the silencing of SENP1 delays the separation of sister chromatids in the metaphase (Nayak and Müller, 2014). DeSUMOylation of HIF-1α by SENP1 under hypoxic conditions is essential for HIF-1α stability and expression of HIF-1α target genes. The recruitment of SENP1 to specific substrates is associated with post-translational modifications, for example, through the phospho-dependent binding of SENP1 to BCL11B (Zhang et al., 2012). SUMOylation and deSUMOylation also control proliferation and aging because the downregulation of *SENP1* expression by RNA interference increased the total SUMOylated proteins, the number of PML bodies and p53-mediated transcriptional activity, and premature aging (Andreou and Tavernarakis, 2010b). SENP1 functions as a novel transcriptional activator mediated by the androgen receptor by deSUMOylation of histone deacetylase 1 (HDAC1). These studies suggest SENP1 plays a main role in carcinogenesis. The overall dynamics of SUMOylation/deSUMOylation may be changed by cell growth, cell cycle conditions, and disease state, and SENP proteins might play an important role in cancer growth and be an appropriate target for cancer treatment and therapy.

Natural compounds, especially phytochemicals, minerals, and vitamins, were also demonstrated to prevent cancer *in vivo* and *in vitro*. In modern medicine, more than 3,000 species of plants have been reported (Millimouno et al., 2014). Many natural compounds have anticancerous and antiturmeric properties, including antioxidative, antiangiogenetic, antiproliferative, and apoptotic properties (Alemi et al., 2013; Rashid et al., 2019; Ahmadi et al., 2020). Prostate cancer cells were killed by *Toona sinensis* leaf extract most likely by induced apoptosis (Chen et al., 2009). Curcumin has been reported to show great therapeutic potential for osteosarcoma by Leow et al. (2010). There are lines of evidence that curcumin contributes to the reduction of cancer cell proliferation by controlling autophagy (e.g., chronic myeloid leukemia, malignant glioma, and esophageal cancer cells) (Jia et al., 2009; Aoki et al., 2007; O'Sullivan-Coyne et al., 2009). Cancer cells derived from melanoma and cervical carcinoma undergo cell death under the influence of oridonin, which exhibits significant antiproliferative activity (Abelson, 1990; Cui et al., 2006). In comparison with various current cancer treatment methods, natural products are cheaper and seem to have fewer side effects.

Overexpression of SENP1 has been detected in various types of cancer. Therefore, it is urgent to identify potent molecules that can bind to SENP1, inhibit SENP1, and can ultimately be used to treat cancer. Previously, we studied some secondary metabolites and observed their effects on SENP1 protein in various cancer cell lines and nervous system cells (Amiraslani et al., 2012; Alemi et al., 2013; Esmaeilzadeh et al., 2013; Rashid et al., 2019; Ahmadi et al., 2020; Taghvaei et al., 2021b; Taghvaei et al., 2021c). Because of the important roles of SENP1 in cancer biology, we sorted the compounds with better properties and more active groups to interact with SENP1, which could be stronger inhibitors for SENP1 and could be proposed as anticancer drugs. However, these compounds were not sufficient for conclusion, and then, we screened the ZINC database with more compounds.

Since traditional drug discovery is based on a random trial-and-error approach, comprehensive screening methods and *in vitro* tests are used to measure the activity of a large number of compounds against a hypothetical target. Moreover, these methods are very expensive and time-consuming. Alternatively, if the three-dimensional structure of the target molecule is known, simulated molecular docking can be a useful tool in facilitating the drug discovery process. This *in silico* method would lead to a cheaper and faster identification of drug candidates. Then, laboratory-based tests and clinical trials can be used to further validate the identified drug candidates (Thomsen and Christensen, 2006).

Molecular docking has been used to predict potent drug molecules that inhibit the growth of cancer stem cells (Stark and Powers, 2011). The results of molecular docking can be effective in advancing potential strategies for drug design (Balupuri et al., 2014). Alterations such as important functional changes in the protein structure, folding of proteins into their natural three-dimensional structures, and various types of interactions between two proteins or between a protein and a candidate drug molecule can occur within the range of a few femtoseconds to a few hundred nanoseconds. A molecular dynamics of such events may involve tens of thousands of atoms, which represents one or more biological macromolecules surrounded by a solvent environment consisting of dissolved ions and a high number of water molecules (Seibert et al., 2005). Computer-based drug design techniques can be effective in reducing the cost and increasing the speed of drug discovery. There are two types of applications of molecular dynamics to confirm a binding method obtained by docking (also called pose). First, molecular dynamics is used as a final filter after docking and high-power ranking. Second, molecular dynamics is used to optimize hits (or leads) (Zhao and Caflisch, 2015). Therefore, it is reasonable to combine the docking and molecular dynamics simulation for the rational design of new drugs. The first part includes the use of computational techniques in the drug design process. The next part is the protein flexibility part, which is the flexibility of the complex. The energy calculation section briefly describes the approaches used to evaluate accurate binding energies, which are followed by molecular dynamics simulations during the docking steps (Alonso et al., 2006).

In this study, we initially docked natural compounds apigenin, aspirin, berberine, catechin, curcumin, cinnamic acid, ethyl gallate, ferulic acid, kaempferol, naringin, naringenin, quercetin, resveratrol, rutin, rusmarinic acid, silybin, silymarin, senegenin, sulforaphane, tangeretin, tannic acid, triptolide, ugonin, urolithin A, urolithin M5, ursolic acid, usnic acid, ugonin, veatric acid, and momordin IC (as a positive control of SENP1) with the SENP1 protein which act as compounds for molecular dynamics simulation (MDS) and were selected based on higher docking free energy and the lowest toxicity. Moreover, we carried out virtual screening of the ZINC database, and the selected compounds with high docking free energy and the lowest toxicity were applied for MDS.

## 2 Material and methods

**FIGURE 1:**
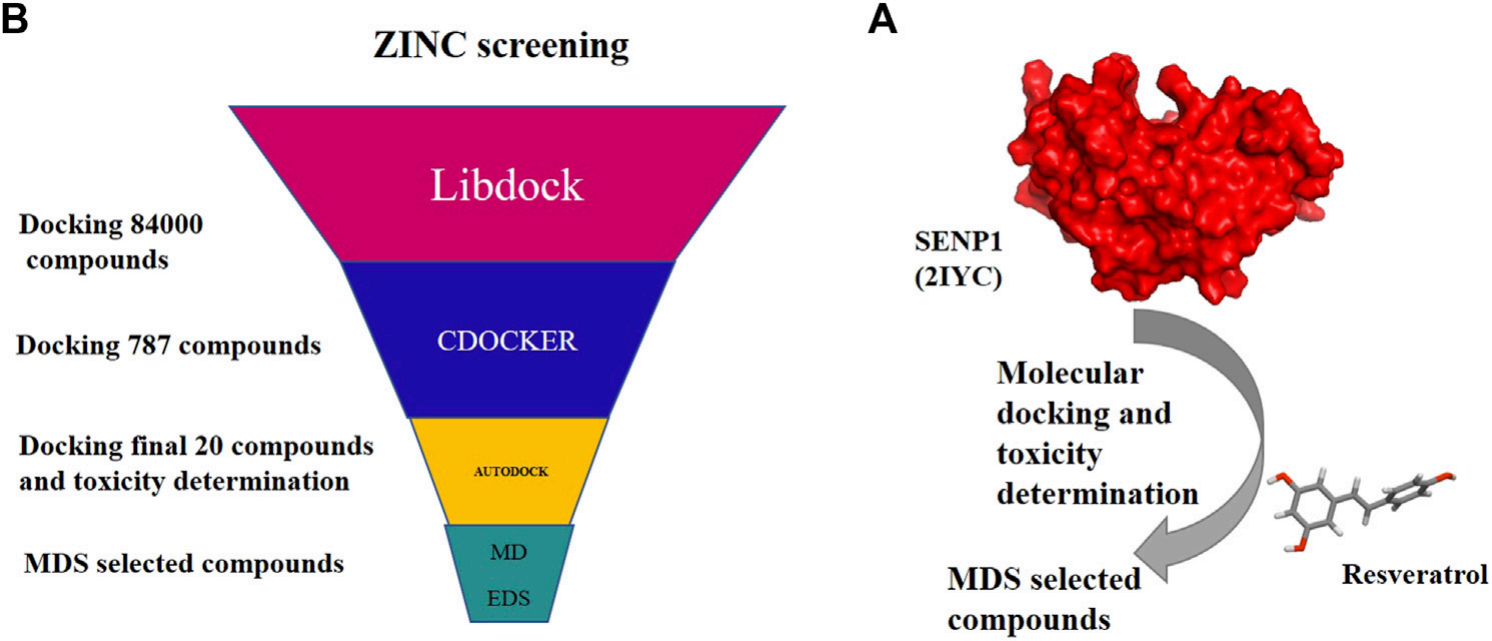
Flow chart of research stes: **(A)** molecular docking and molecular dynamics of suggested compounds and **(B)** virtual screening of the ZINC library with 84,000 compounds.

To survey 28 natural compounds, we selected a suitable protein structure based on the resolution and amino acids of the binding site. Initially, the SENP1 protein and natural compounds were prepared using Molegro Virtual Docker and the SENP1 active site was determined. Then, molecular docking was carried out between SENP1 and the compounds. The compounds were also evaluated by Lipinski’s rule of five, and ADMET and TOPCAT modes. Then, the selected compounds underwent MDS and essential dynamic analysis, as shown in Figure 1A.

### 2.1 Molecular docking

#### 2.1.1 Protein and ligand preparation

Protein information was extracted from the UniProt database (http://www.uniprot.org/) (Consortium, 2014), and the protein structure (PDB ID: 2IYC) was extracted from the RCSB database (https://www.rcsb.org) (Rose et al., 2016). Referring to drug databases (http://zinc.docking.org/) (Irwin et al., 2012) and PubChem (http://pubchem.ncbi.nlm.nih.gov) (Kim et al., 2015), natural compounds were also downloaded. Before molecular docking, the protein and ligand structures were prepared by Molegro Virtual Docker.

Molecular docking of SENP1 was performed with natural compounds including apigenin, aspirin, berberine, catechin, curcumin, cinnamic acid, ethyl gallate, ferulic acid, kaempferol, naringin, naringenin, quercetin, resveratrol, rutin, rusmarinic acid, silybin, silymarin, senegenin, sulforaphane, tangeretin, tannic acid, triptolide, ugonin, urolithin A, urolithin M5, ursolic acid, usnic acid, ugonin, veatric acid, and momordin IC using AutoDock software (Morris et al., 2009). The binding position included the coordinates -x center = 33.658, -y center = −16.605, and -z center = −0.55 with amino acids TRP465, LEU466, HIS529, GLY531, VAL532, HIS533, TRP534, MET552, GLN596, and CYT603.

Molecular docking of the ligands with SENP1 was performed by AutoDock.4. Polar hydrogen atoms were incorporated, and non-polar hydrogens were merged. Gasteiger charges were added. Docking protocol was performed in a grid box consisting of 60 × 60 × 60 (x, y, and z) points at the center and with the grid resolution of 0.375 Å to cover the SENP1-binding site. Docking was performed with a genetic algorithm. Then, 25×10^5^ energy evaluations with the maximum of 27,000 generation numbers were performed in this simulation. The population size was fixed at 150 in each run, mutation rate at 0.02, and crossover rate at 0.80. For the ligands, the torsions were defined using the “Ligand torsions” menu option of AutoDock4. Other parameters were set to default amounts (Taghvaei et al., 2021b). Discovery Studio V16.1.0.15. was used to analyze docking results that were obtained from AutoDock.4. For MDS and essential dynamic, selected docking complexes with the lowest side effects were applied.

### 2.2 Investigation of toxicity by an *in silico* method

After molecular docking, it is necessary to investigate the toxicity effects of these compounds. When a chemical compound is used as an oral drug, it first enters the stomach. The drug must be resistant to the vicinity of gastric acid and must be able to enter intestinal cells after passing through the stomach. The drug must be able to enter the bloodstream through the intestinal wall and go through the blood vessels to the liver. In the liver, the drug must show resistance to metabolism, until it eventually enters the bloodstream and reaches the target. It is observed that a drug whose computational and laboratory effects were verified on the target protein may not be suitable for using as an oral drug because this complex route may cause changes in its pharmacological properties. On the other hand, about 20 years ago, before the introduction of computational methods, about 50% of potential therapeutic compounds failed before entering the clinical stage. Therefore, a successful drug is not necessarily the best inhibitor of its purpose. It needs to meet the necessary criteria such as absorption, distribution, metabolism, excretion, and toxicity (as indicated by ADMET) and TOPKAT that is based on QSAR and QSTR models.

#### 2.2.1 ADMET

The human body is constantly exposed to multiple compounds over time. During development, several defense barriers have been created to inactivate them, including the family of cytochrome P450 isoenzymes (CYP) present in the liver, the active return of drug compounds using permeability glycoprotein (P-gp), the blood–brain barrier, and the glomerular filtration assembly in kidneys. Because the range of chemical compounds is so wide, it is almost impossible to examine each of them in the laboratory. However, further investigation can be assisted by the QSAR and QSTR methods. The basis of these methods is based on the principle that compounds with similar structures will have similar functions (Cheng et al., 2012).

#### 2.2.2 TOPCAT

As explained, the ADMET method is based on a training series, so its operation is dependent on an experimental database. However, in the TOPCAT method, the effects of different chemical compounds at different concentrations were measured *in vitro* for a period of 2 years on mice and rats. The TOPKAT model is a tool for using these data as training series and using QSTR methods and finding similar structures in the newly introduced structure to predict the toxic properties of new compounds.

### 2.3 Molecular dynamics simulation

#### 2.3.1 MD simulation and binding free energy prediction

The GROMACS 4.6.5 package was run on a great-performance Linux cluster to distinguish the behavior of ligands with GROMOS53a6 force field (Van Der Spoel et al., 2005). The PRODRG server was used to obtain the GROMACS coordinates and ligand topology parameter files. In this process, the SENP1–ligand complexes were immersed in a dodecahedron-modeled box (x, y, and z) with 238.58 nm^3^. SPC/E water molecules were added to solvate the system. Solvation was performed with 1 nm of the distance between the cage edges and the protein periphery. Then, system neutralization was accomplished by the addition of seven chloride ions. To inhibit the instability of the MDS, the solvated system was subjected to 1,000-cycle minimization. Before MDS, the temperature of the crystal structure reached 300 K and was balanced during 100 ps at the conditions of constant volume and temperature (NVT). Then, the system was changed to a constant pressure and temperature (NPT) and was balanced for 100 ps. The non-bonded cut-off was fixed at 10 Å, and the non-bonded pair list was updated every five steps. MDS was performed via the particle mesh Ewald (PME) parallel version in GROMACS. The LINKS mode was used to impose all hydrogen bonds and motion equation integration (Essmann et al., 1995), and structural snapshots were flushed every 500 steps (1 ps) (Van Der Spoel et al., 2005). We performed 100-ns MDS between the best compounds and SENP1 during 50 ×10^6^ steps and free-SENP1. At the end, we utilized GRACE software for plotting (http://plasma-gate.weizmann.ac.il/Grace/).

#### 2.3.2 Molecular mechanics–Poisson-Boltzmann surface area (MM-PBSA)

To count the binding energy of biomolecular complexes, MM-PBSA was extensively utilized and its combination with the MDS is an effective manner to study biomolecular interactions. Other uses of g_mmpbsa include the better potency to differentiate inactive and active lead molecules, rescoring of docked assemblies, and decomposing of the total counted binding energy into portions per residue (Massova and Kollman, 2000; Safi and Lilien, 2012; Andreoli and Del Rio, 2015). The MM-PBSA mode accomplished in the GROMACS plan was used to calculate the difference of free energies (ΔG) between natural compound configurations and SENP1.

#### 2.3.3 Analysis of molecular dynamics trajectories

To survey structural alternations in SENP1, we analyzed the trajectory file of compounds for achieving the results such as the root-mean-square deviation (RMSD), root-mean-square fluctuation (RMSF), hydrogen bond (Hb), radius of gyration (Rg), solvent-accessible surface area (SASA), dictionary of the secondary structure of protein (DSSP), and minimum distance (mindist) using g_rmsd, g_rmsf, g_hbond, g_gyrate, g_sasa, do_dssp, and *g_mindist* built-in functions of the GROMACS package, respectively. Interactions between SENP1 and ligands were portrayed in Discovery Studio (Studio, 2008).

### 2.4 Essential dynamics

Essential dynamics, known as the principal component analysis (PCA), can show the collective atomic motion of compound–SENP1 complexes by the GROMACS tool. PCA was computed using g_covar and g_anaeig built-in functions of the GROMACS package. PCA is a standard protocol for the characterization of eigenvectors and the projection across the first PC1 and PC2 (Taghvaei et al., 2021d).

### 2.5 Virtual screening of the ZINC database

For virtual screening, Figure 1B, the LibDock algorithm from Discovery Studio was first used to screen a library of 84,000 natural compounds from the ZINC database (IBScreen NP). LibDock is a hard docking program that calculates protein hotspots using a grid located at the active site using polar and non-polar probes. Therefore, sensitive points are used to connect the ligands to the binding site for favorable interaction. PDB ID: 2IYC of SENP1 was prepared for docking through the LibDock algorithm in Discovery Studio. Considering the amino acids TRP465, LEU466, HIS529, GLY531, VAL532, HIS533, TRP534, MET552, GLN596, and CYT603, the binding site was determined. Protein and ligands were prepared by setting a 100 number of sensitive points. Overall screening was performed with LibDock; at the end, the compounds were scored based on the LibDock score, and compounds with the LibDock score higher than momordin, as control (97.64), (787 compounds), were docked to SENP1 with CDOCKER. CDOCKER is an implementation of a CHARMm-based docking tool. The binding site was determined similar to the LibDock method, protein and ligands were selected, and top hits were set to 100. Docking was then performed with CDOCKER, and finally, the resulting compounds were sorted based on CDOCKER energy, and the top 20 compounds with CDOCKER energy were selected for molecular docking with AutoDock.4.

A total of 20 compounds obtained from the virtual screening of 84,000 natural compounds of the ZINC database, according to CDOCKER energy, were docked with SENP1 using AutoDock.4, according to molecular docking instructions (section 2.1 of the present study). Toxicity of the 20 compounds was measured by Lipinski’s law, and ADMET and TOPCAT criteria. Then, the desired compounds in terms of binding energy of AutoDock.4, CDOCKER energy, ADMET, and TOPCAT were extracted. Finally, three compounds were selected and applied for further studies with molecular dynamics.

Here, molecular dynamics was performed for 100 ns for further investigation of selected compounds (ZINC79204151, ZINC80680876, and ZINC79204151), according to the Molecular Dynamics Instruction (section 2.2 of the present study). We also applied PCA to these compounds (according to section 2.4 of the present study).

## 3 Results

### 3.1 Molecular docking study

We performed flexible docking using AutoDock.4 so that the optimal ligand geometry was defined in docking. Furthermore, the present molecular docking studies could contribute to further development and understanding of SENP1 inhibitors for the prevention of cancer. The compounds are presented according to their binding energy in Table 1. Aspirin, berberine, cinnamic acid, ferulic acid, resveratrol, and momordin with binding energies −5.79, −6.65, −4.89, −6.21, −5.91, and −9.53 kcal/mol, respectively, were applied to MDS and more surveys. We proposed these compounds inhibit SUMO1 binding to SENP1, as was previously suggested (Guo and Zhou, 2016).

**TABLE 1:** Free binding energy of natural compounds using AutoDock4.

Free binding energy using AutoDock4 (kcal/mol)	Compound name	Number
−6.65	Berberine	1
−6.57	Usnic acid	2
−6.33	Curcumin	3
−6.32	Naringenin	4
−6.22	Triptolide	5
−6.21	Ferulate	6
−6.04	Ursolic acid	7
−5.99	Senegerin	8
−5.91	Resveratrol	9
−5.89	Apigenin	10
−5.86	Tangeretin	11
−5.79	Aspirin	12
−5.6	Veratric acid	13
−5.38	Kaempferol	14
−5.33	Rusmarinate	15
−5.23	Silibinin	16
−5.07	Silymarin	17
−4.89	Cinnamic acid	18
−4.86	Urolithin A	19
−4.85	Catechin	20
−4.77	Ugonin	21
−4.7	Quercetin	22
−4.49	Urolithin M5	23
−3.87	Naringin	24
−3.68	Ethyl gallate	25
−3.68	Rutin	26
−3.67	Sulforaphane	27
+8.52	Tannic acid	28
−9.53	Momordin	Control

### 3.2 Investigation of toxicity by an *in silico* method

Drug-likeness is a qualitative meaning used in the design of a drug, indicating how a substance is “drug-like”. The drug-likeness properties of these compounds were gained using Lipinski’s rule of five, ADMETSAR, and TOPCAT. All of the compounds obeyed Lipinski’s rule of five. The results of the drug-likeness by ADMET and TOPKAT are presented in Supplementary Tables S1, S2. The chemical properties of the identified compounds require the determination of pharmacokinetic properties evaluated in terms of absorption, distribution, metabolism (how to interact with cytochromes), excretion (excretion of the kidney), and toxicity. AMES carcinogenicity was safe for all of the compounds, and they were neither a carcinogen nor a mutagen. The results of ADMET also showed all compounds are able to cross the blood–brain barrier and the intestinal wall but that ferulic acid cannot cross the blood–brain barrier and berberine cannot cross the intestinal wall. Then, the compounds that cross the intestinal wall and blood–brain barrier can easily be absorbed by the intestine to enter the liver and be applied for brain tumors, respectively. All the compounds were permeable to CaCO_2_. All compounds could be localized in the mitochondria except cinnamic acid, which could be localized in the plasma membrane.

Another indicator that was evaluated at this stage was the ability to bind and suppress glycoproteins that are actively involved in the removal of xenobiotics from the cell. The ideal drug-like compounds are compounds that do not bind to glycoproteins and, therefore, do not leave the cell. In this case, five compounds were neither substrates for glycoproteins nor inhibitors. The point to be considered is that from these selected compounds, ideal compounds are neither glycoprotein substrates nor inhibitors. Because these glycoproteins play other roles that by inhibiting them, these roles can be inhibited and the normal function of the cell is likely disturbed.

Another indicator that has been measured is the possibility of metabolizing by CYP450 and inhibiting this complex of metabolic proteins. Ferulic acid, cinnamic acid, and aspirin were neither CYP450 substrates nor inhibitors, but berberine was a CYP3A4 substrate and a CYP1A2 and CYP2D6 inhibitor. Similarly, resveratrol was not a substrate but was an inhibitor of CYP1A2, CYP3A4, CYP2C9, and CYP2C19. Fish toxicity (FHMT) was high for all compounds except berberine. *Tetrahymena pyriformis* toxicity (TPT) was high for all compounds, and honey bee toxicity (HBT) was high for all compounds except berberine. Moreover, all the compounds represented weak inhibition potential of the human ether-a-go-go-related gene (hERG), whose expression plays a significant role in the repolarization of the cardiac action potential (Sanguinetti et al., 1995).

On the other hand, to evaluate the toxicity of the identified compounds based on the QSTR model, TOPKAT (Discovery Studio 2.5, version 16, Biovia, San Diego, CA, United States) was applied. This model is based on repetitive statistical methods with high credit ratings and highly developed. In this model, the toxic effects of these compounds based on their chemical structure were predicted. The TOPCAT results showed all compounds are safe for the AMES mutagenicity except cinnamic acid. In addition, in all the National Toxicology Program (NTP) carcinogenicity tests, berberine was carcinogenic. Berberine and aspirin were also carcinogenic only in the NTP Carcinogenicity Call (male rat) (v3.2) test, cinnamic acid was carcinogenic only in the NTP Carcinogenicity Call (female rat) (v3.2) test, and ferulic acid was carcinogenic only in the NTP Carcinogenicity Call (female mouse) (v3.2) test, while resveratrol was not carcinogenic in any test. The Food and Drug Administration (FDA) carcinogenicity tests emphasize the contact frequency, and long-term effects faced the compounds under investigation. Berberine was not carcinogenic in any FDA carcinogenicity test. Resveratrol and aspirin were carcinogenic only in the FDA Carcinogenicity Male Rat Non *vs.* Carc (v3.1) test. Cinnamic acid was also carcinogenic only in the FDA Carcinogenicity Male Rat Single *vs.* Mult (v3.1), FDA Carcinogenicity Male Mouse Single *vs.* Mult (v3.1), and FDA Carcinogenicity Female Mouse Non *vs.* Carc (v3.1) tests. Ferulic acid was also carcinogenic only in the FDA Carcinogenicity Male Rat Non *vs.* Carc (v3.1), the FDA Carcinogenicity Male Rat Single *vs.* Mult (v3.1), and FDA Carcinogenicity Female Mouse Single *vs.* Mult (v3.1) tests.

Developmental toxicity potential indicates mutagenic characteristics during development that can restrict their use in pregnancy; non compounds were mutagenic except for berberine. The skin irritation test (v6.1) showed only berberine cannot irritate the skin. Skin sensitization examination revealed berberine, resveratrol, and aspirin do not cause skin allergies in the Sensitization NEG v SENS (v6.1) test, and all compounds cause skin allergies through the Skin sensitization MLD/MOD v SEV (v6.1) test. The ocular irritancy test also showed only resveratrol and aspirin cannot cause ocular irritation through the Ocular irritancy SEV/MOD *vs.* MLD/NON (v5.1) test. Through the Ocular Irritancy SEV *vs.* MOD (v5.1) test, aspirin, berberine, and ferulic acid cannot cause ocular irritation and through the Ocular Irritancy MLD *vs.* NON (v5.1) test, only aspirin and ferulic acid do not cause ocular irritation. In the aerobic biodegradability test, berberine, cinnamic acid, and ferulic acid were biodegradable, which means these compounds can be biodegraded by air. We concluded using TOPCAT and ADMET properties, resveratrol and aspirin have the lowest carcinogeneses and toxicity properties. These five compounds showed most favorable factors, for example, lower carcinogenicity and mutagenicity, crossing the blood–brain barrier and intestinal wall, penetration to CaCO_2_, no inhibition of CYP enzymes and glycoprotein, and aerobic biodegradability.

### 3.3 MD simulation

The simulation of physical interactions and moves of molecules and atomic systems by the computer simulation method known as MDS was carried out. Affinity and binding mechanisms of compounds to the SENP1 at an atomistic scale were applied to explore binding free energy prediction. Since aspirin, berberine, cinnamic acid, ferulic acid, and resveratrol had high binding energy and lowest toxicity, they were selected for further steps.

Analogical analysis of structural aberrations in free-SENP1 and ligand–SENP1 complexes such as RMSD, RMSF, H-bond, Rg, SASA, DSSP, and mindist was calculated.

#### 3.3.1 Free energy calculation

The average binding energy for different ligands was computed, which includes 35.791 for aspirin, −3.918 for berberine, 197.111 for cinnamic acid, 441.350 for ferulic acid, −82.829 for resveratrol, and −455.685 kcal/mol for momordin.

#### 3.3.2 Structural deviations and compactness

**FIGURE 2:**
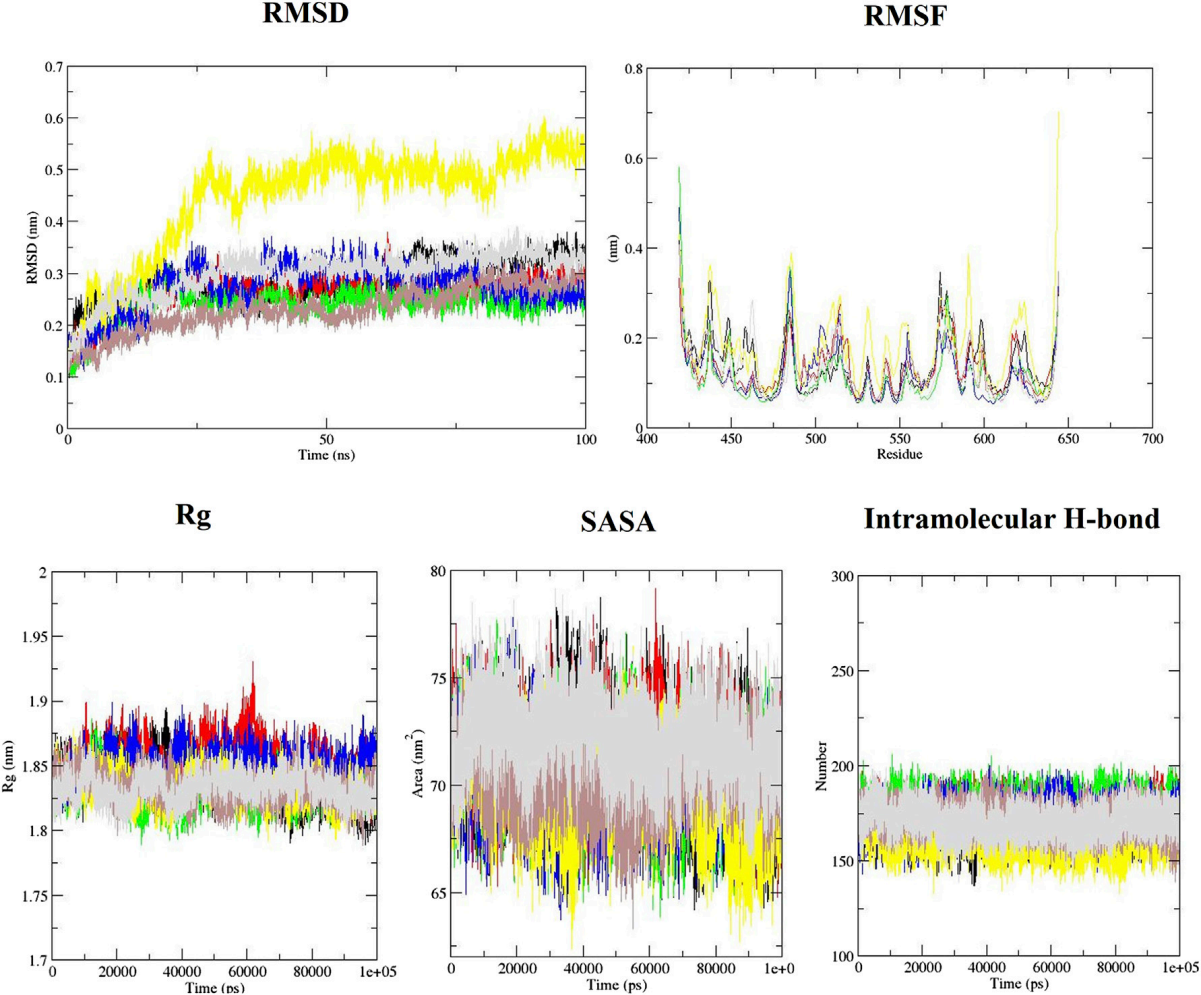
RMSD, RMSF, Rg, SASA, and intramolecular hydrogen bond plots of free-SENP1 and SENP1 complexes, (black) free-SENP1, (red) aspirin, (green) berberine, (blue) cinnamic acid, (yellow) ferulic acid, (brown) resveratrol, and (gray) momordin.

The behavior of the ligands during simulation was evaluated through the RMSD (Figure 2). RMSD was used to measure ligand stability during simulation (Taghvaei et al., 2021b). Average RMSD for aspirin, berberine, cinnamic acid, ferulic acid, resveratrol, momordin, and free-SENP1 were calculated to be 0.27, 0.24, 0.27, 0.45, 0.23, 0.28, and 0.3 nm, respectively, as shown in Figure 2. We observed berberine and resveratrol with the lowest average RMSD.

RMSF of the compound–SENP1 complexes and free-SENP1 amino acids are shown in Figure 2. The RMSF was plotted to test the conformational drift seen in RMSD plots and how the compounds affect the dynamic behavior of the amino acids (Taghvaei et al., 2022). RMSF plots showed that most of SENP1–compound complexes have higher stability than free-SENP1 and the most fluctuations are in the N-terminal end, as shown in Figure 2. Ferulic acid demonstrated most fluctuations, while resveratrol indicated the lowest fluctuations. It can be seen that the presence of resveratrol minimizes the major backbone fluctuations and makes a stable structure.

Rg is an indicator of the level of structure compaction, i.e., the polypeptide is unfolded or folded (Taghvaei and Saremi, 2022). The Rg plots for the backbone atoms of protein in the absence of all ligands and in their presence are displayed in Figure 2. It can be seen that the Rg of SENP1 frequently decreases upon binding of the compounds compared with free-SENP1, implying a more compact structure after the simulation. The average Rg values for aspirin, cinnamic Acid, berberine, ferulic acid, resveratrol, momordin, and free-SENP1 were calculated to be 1.85, 1.83, 1.85, 1.83, 1.83, 1.84, and 1.83 nm, respectively, and are shown in Figure 2.

#### 3.3.3 Hydrogen bond analysis

**FIGURE 3:**
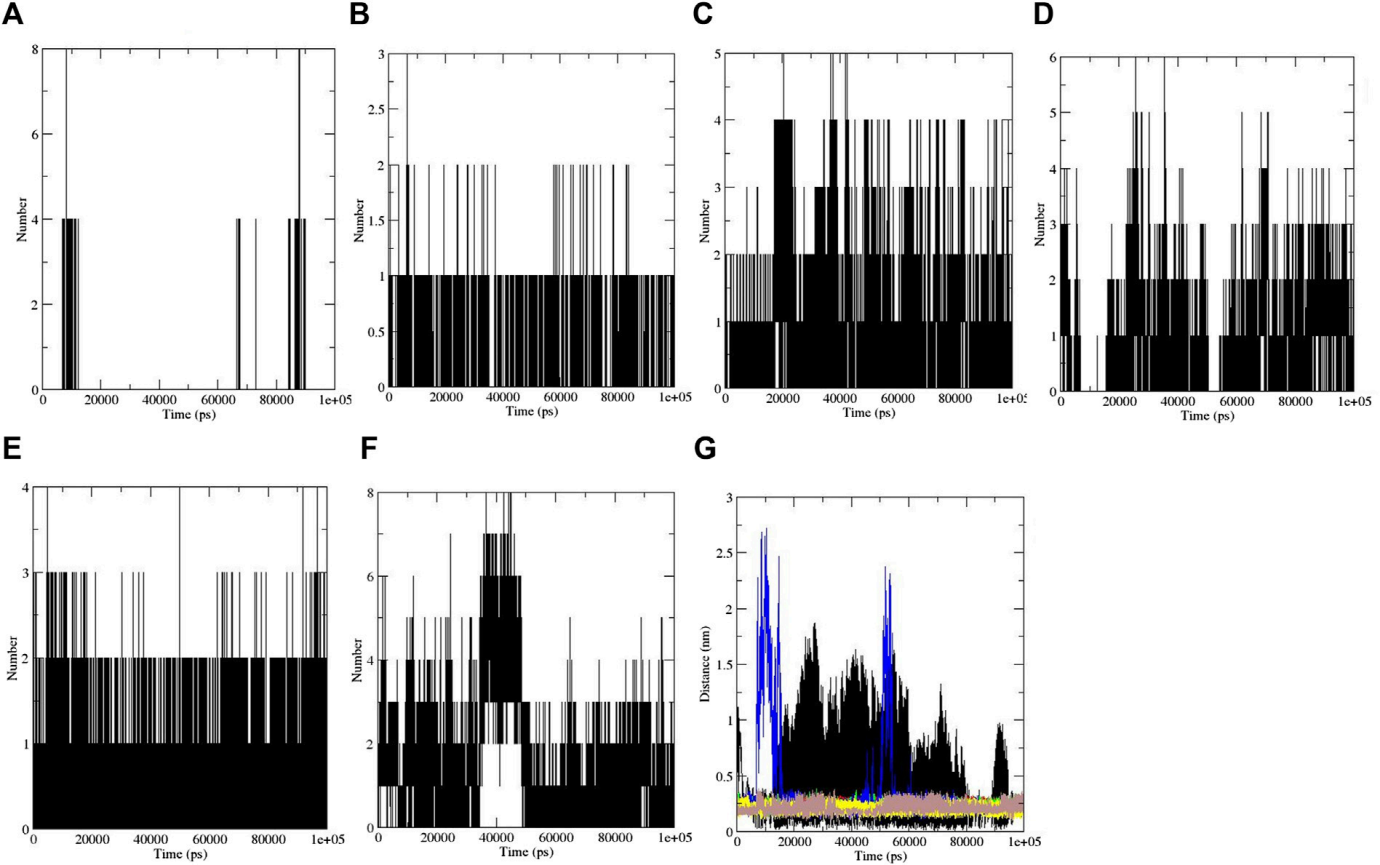
Intermolecular hydrogen bonds: **(A)** aspirin, **(B)** berberine, **(C)** cinnamic acid, **(D)** ferulic acid, **(E)** resveratrol, and **(F)** momordin, and **(G)** distance plots of SENP1 complexes, (black) free-SENP1, (red) aspirin, (green) berberine, (blue) cinnamic acid, (yellow) ferulic acid, (brown) resveratrol, and (gray) momordin.

Furthermore, hydrogen bonding is a factor that plays a major role in maintaining the protein stable conformation (Taghvaei et al., 2021d). To realize the reason of flexibility between the compounds, we performed the NH bond analysis of ligand–protein during simulations which are plotted in Figures 3A–F. The protein–ligand intermolecular hydrogen bond included aspirin: no hydrogen bond, berberine: one hydrogen bond, cinnamic acid: three to four hydrogen bonds, ferulic acid: three hydrogen bonds, resveratrol: two to three hydrogen bonds, and momordin: three to five hydrogen bonds, and is shown in Figures 3A–F.

We also computed the intramolecular hydrogen bond with 174, 179, 173, 161, 171, and 168 hydrogen bonds for aspirin, berberine, cinnamic Acid, ferulic acid, resveratrol, momordin, and free-SENP1, respectively, as shown in Figure 2.

#### 3.3.4 Solvent-accessible surface area

The estimation of SASA provides information about the conformational changes in protein upon ligand binding (Taghvaei et al., 2022). The average SASA for aspirin, resveratrol, and free-SENP1 was found to be 71 nm^2^, the average SASA for berberine, cinnamic acid, and ferulic acid was found to be 70 nm^2^, and the average SASA for momordin was 72 nm^2^, as shown in Figure 2. The differences were not significant.

#### 3.3.5 Secondary structure alterations upon ligand binding

**FIGURE 4:**
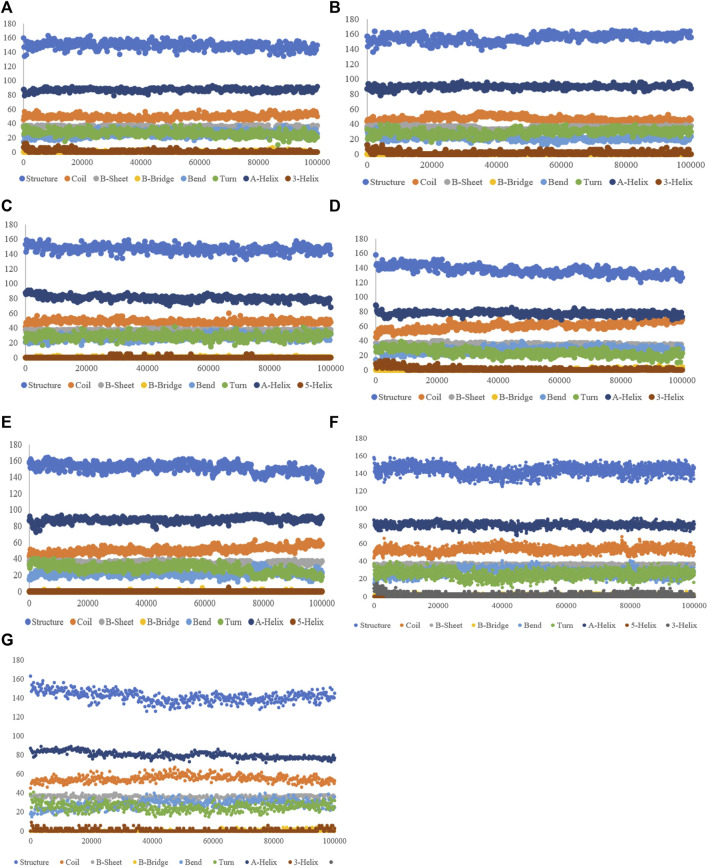
Secondary structure plots of compounds: **(A)** aspirin, **(B)** berberine, **(C)** cinnamic acid, **(D)** ferulic acid, **(E)** resveratrol, **(F)** momordin, and **(G)** free-SENP1.

The purpose of this analysis is measuring the changes in the secondary structure of SENP1 upon binding to our compounds as a function of time. We observed the lowest of alterations in the resveratrol complex including an increase in the α-helix and 3-helix, and a decrease in bend. Most of the alterations are seen in ferulic acid, as shown in Figure 4.

#### 3.3.6 Distance between SENP1 and compounds

The distance between SENP1 and ligands was obtained using the embedded packages within GROMACS. The distance between aspirin, berberine, cinnamic acid, ferulic acid, resveratrol, and momordin were 0.43, 0.26, 0.22, 0.37, 0.22, and 0.21 nm, respectively, as shown in Figure 3G. Cinnamic acid and resveratrol demonstrated the lowest distance with SENP1 protein.

### 3.4 Analysis of the ligand–protein interaction

**FIGURE 5:**
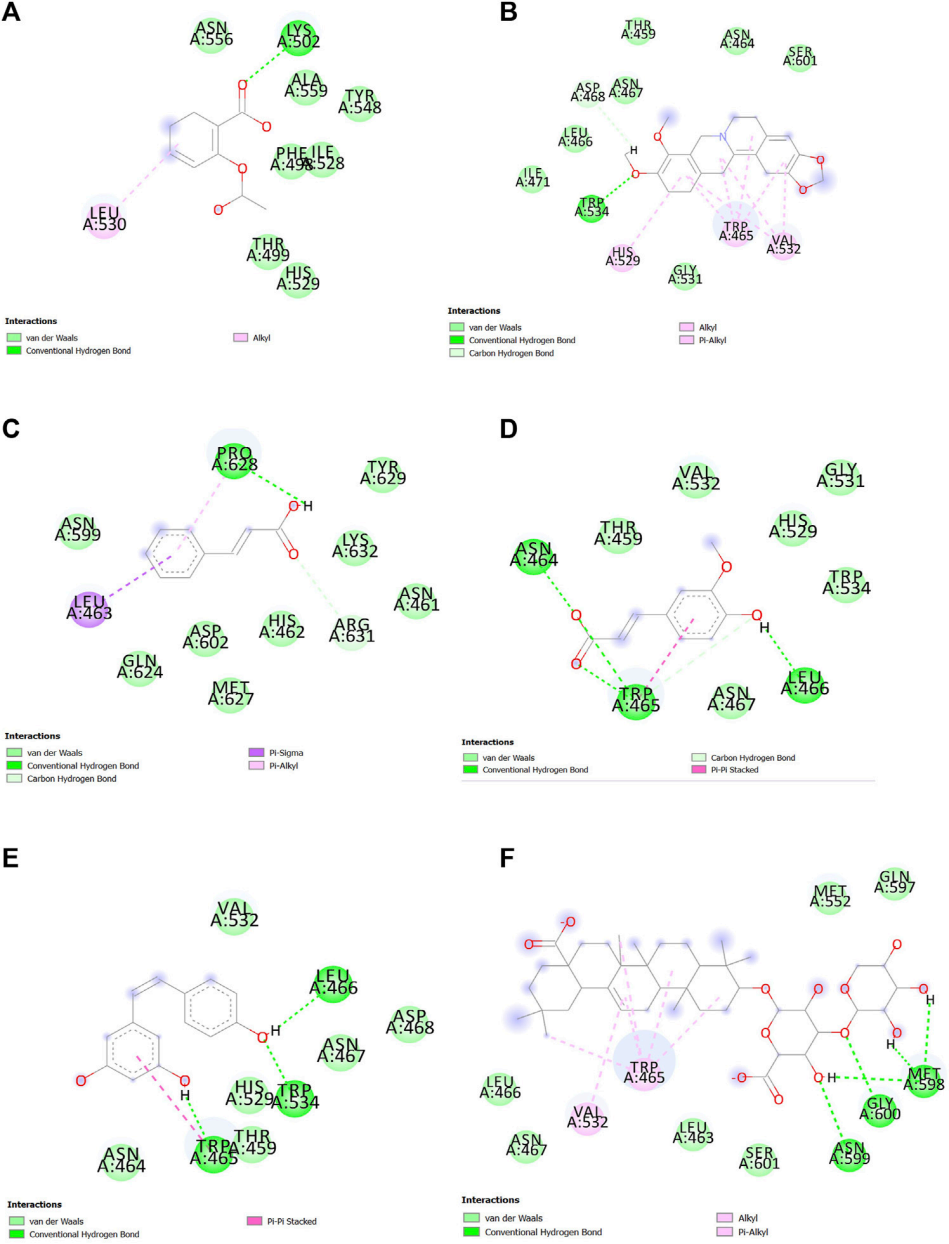
Interactions between **(A)** aspirin, **(B)** berberine, **(C)** cinnamic acid, **(D)** ferulic acid, **(E)** resveratrol, and **(F)** momordin individually with SENP1 (2D structures) in molecular docking by Discovery Studio.

**FIGURE 6:**
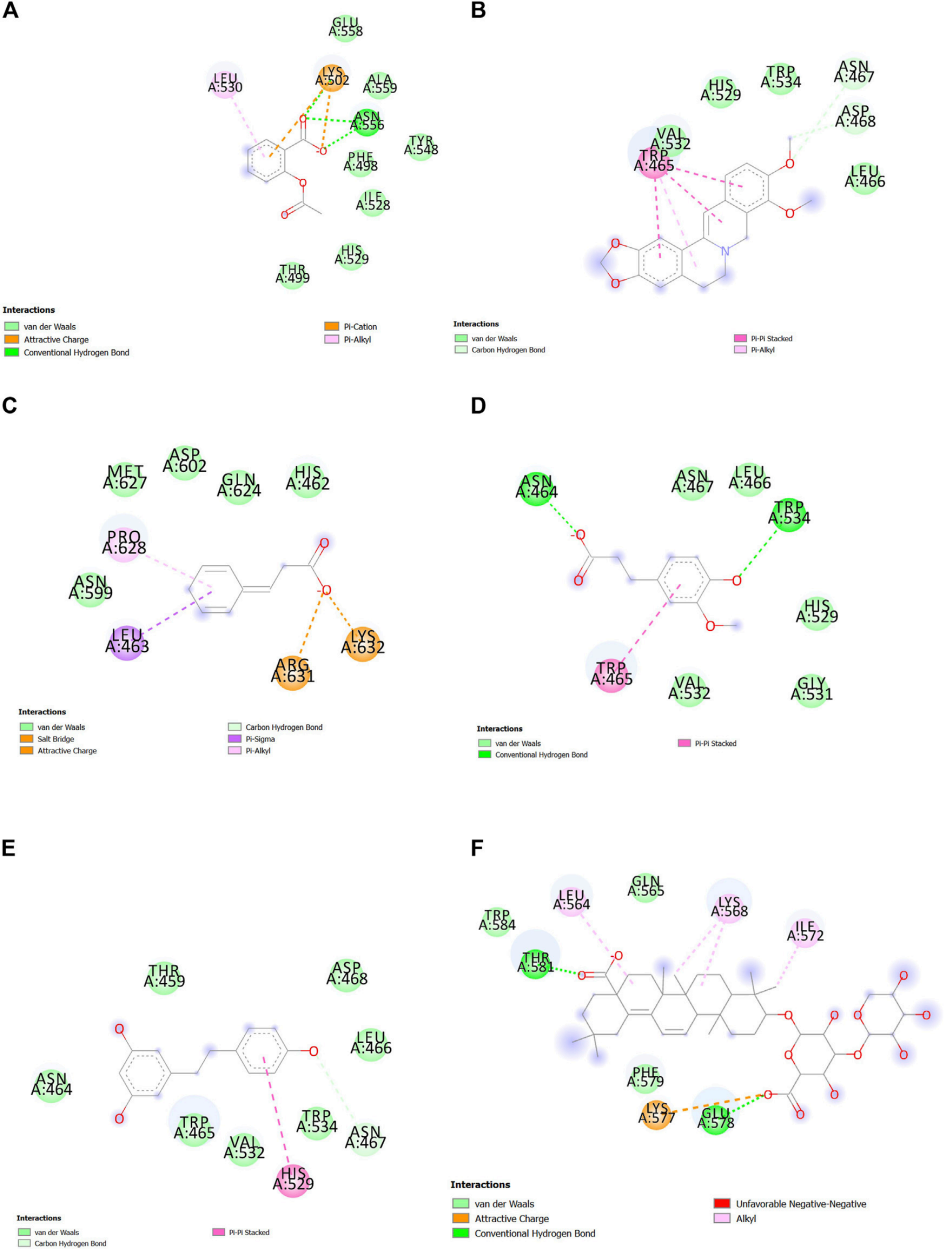
Interactions between **(A)** aspirin, **(B)** berberine, **(C)** cinnamic acid, **(D)** ferulic acid, **(E)** resveratrol, and **(F)** momordin individually with SENP1 by Discovery Studio after molecular dynamics simulation (2D structures).

After docking and MDS, the interaction between ligands and SENP1 was visualized and analyzed using Discovery Studio, as shown in Figure 5; Figure 6, respectively. Two-dimensional shapes represent how the compounds are located in the crystallographic structure. The most observed interactions were van der Waals interactions, and amino acids of the active site involved in these interactions and hydrogen interactions included TRP465, LEU466, HIS529, GLY531, VAL532, and TRP534. Most hydrogen bonds were observed in ferulic acid and resveratrol.

### 3.5 Essential dynamics analysis

**FIGURE 7:**
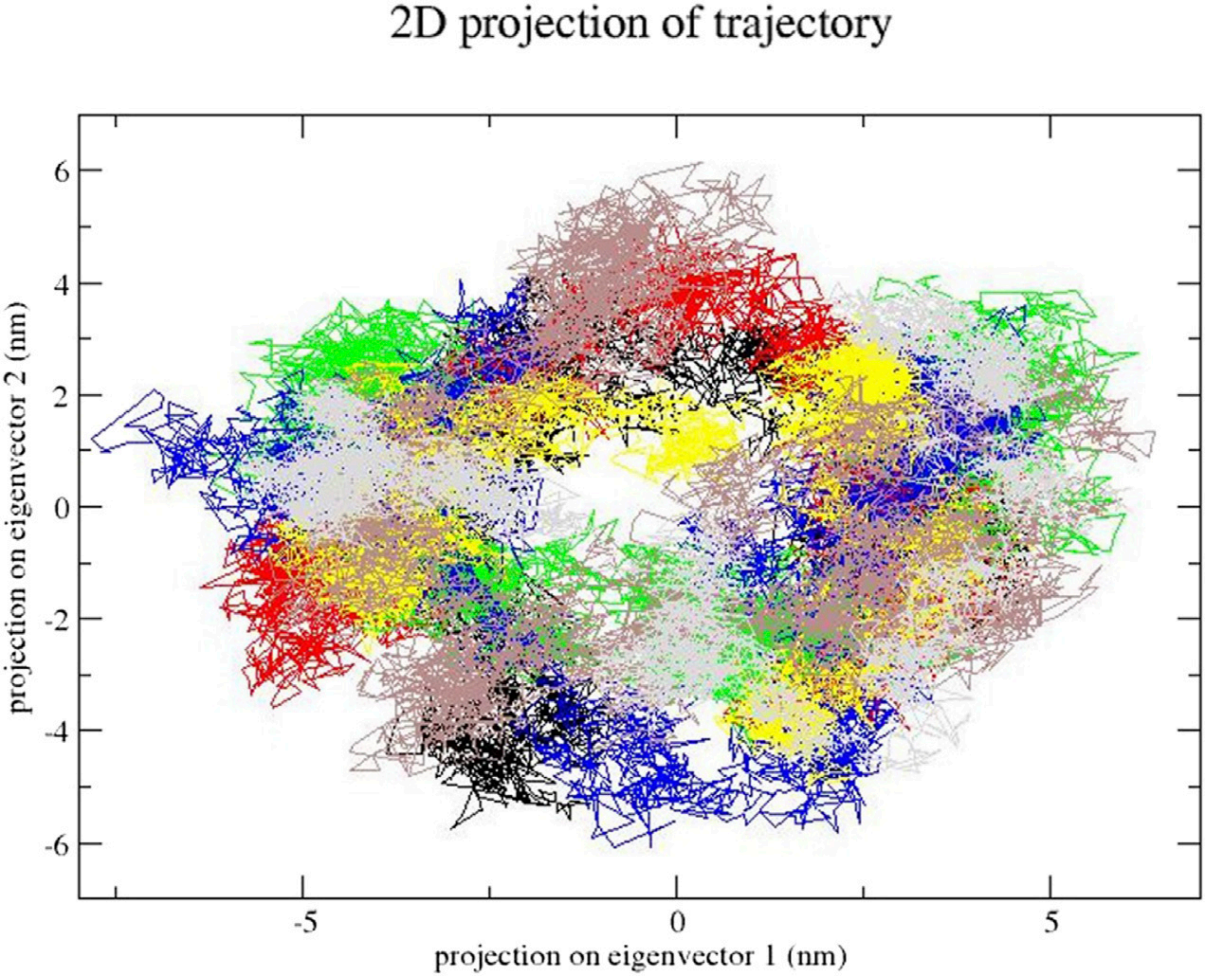
Principal component analysis. Projection of the motion for compounds: (black) free-SENP1, (red) aspirin, (green) berberine, (blue) cinnamic acid, (yellow) ferulic acid, (brown) resveratrol, and (gray) momordin.

In this step, we used essential dynamics analysis to obtain the dynamics of various compound complexes. The projection of trajectories of compound–SENP1 complexes during essential dynamics in the phase space along the first two principal components (PC1 and PC2) at 300 K is plotted in Figure 7. It predicts the large-scale collective motions for the compounds and free-SENP1. PCA showed that, due to ligand binding, the structural dynamics is changing. The Figure 7 plot clearly indicates most compounds occupied less space in the phase space than free SENP1. We observed a decrease in flexibility of complex compounds.

### 3.6 Virtual screening of the ZINC database

For virtual screening, in the first stage, the LibDock module from Discovery Studio was used, in which the resulting compounds were sorted according to the LibDock score, and these compounds with a LibDock score greater than or equal to those of momordin were selected. A total of 787 compounds were docked through CDOCKER. Then, the compounds were selected based on CDOCKER energy. A total of 20 compounds were selected to be docked with AutoDock, as shown in Table 2.

**TABLE 2:** Binding energy of the top 20 virtual screening results in molecular docking with AutoDock4.

Number	Compound name	CDOCKER energy	Free energy using AutoDock4
1	ZINC85867188	−115.298	−5.35
2	ZINC08792480	−96.591	−3.82
3	ZINC15894186	−95.83	−4.55
4	**ZINC33916875**	−88.03	−7.91
5	ZINC79210175	−86.6	3.6
6	ZINC85889031	−85	−7.53
7	ZINC79216732	−82	0.68
8	ZINC12891846	−79	−3.88
9	ZINC79216723	−77.48	2.12
10	ZINC35361287	−77	−5.96
11	ZINC85866695	−76.43	−3.78
12	**ZINC79204151**	−75.22	−6.29
13	ZINC70680876	−75	−5.24
14	ZINC79216729	−72.5	1.79
15	ZINC85867865	−72	−4.58
16	ZINC70692063	−72	−4.75
17	ZINC70670218	−68	−6.2
18	ZINC09357104	−67.02	493.05
19	ZINC70666114	−65.39	−5.03
20	ZINC85902334	−65	−5.59

These bold values are the compounds from virtual screening with lowest docking energy and lowest toxicity.

The compounds ZINC33916875, ZINC85902334, and ZINC79204151 had higher binding energy and lowest toxicity than the others.

#### 3.6.1 Analysis of ligand–protein interactions in molecular docking

**FIGURE 8:**
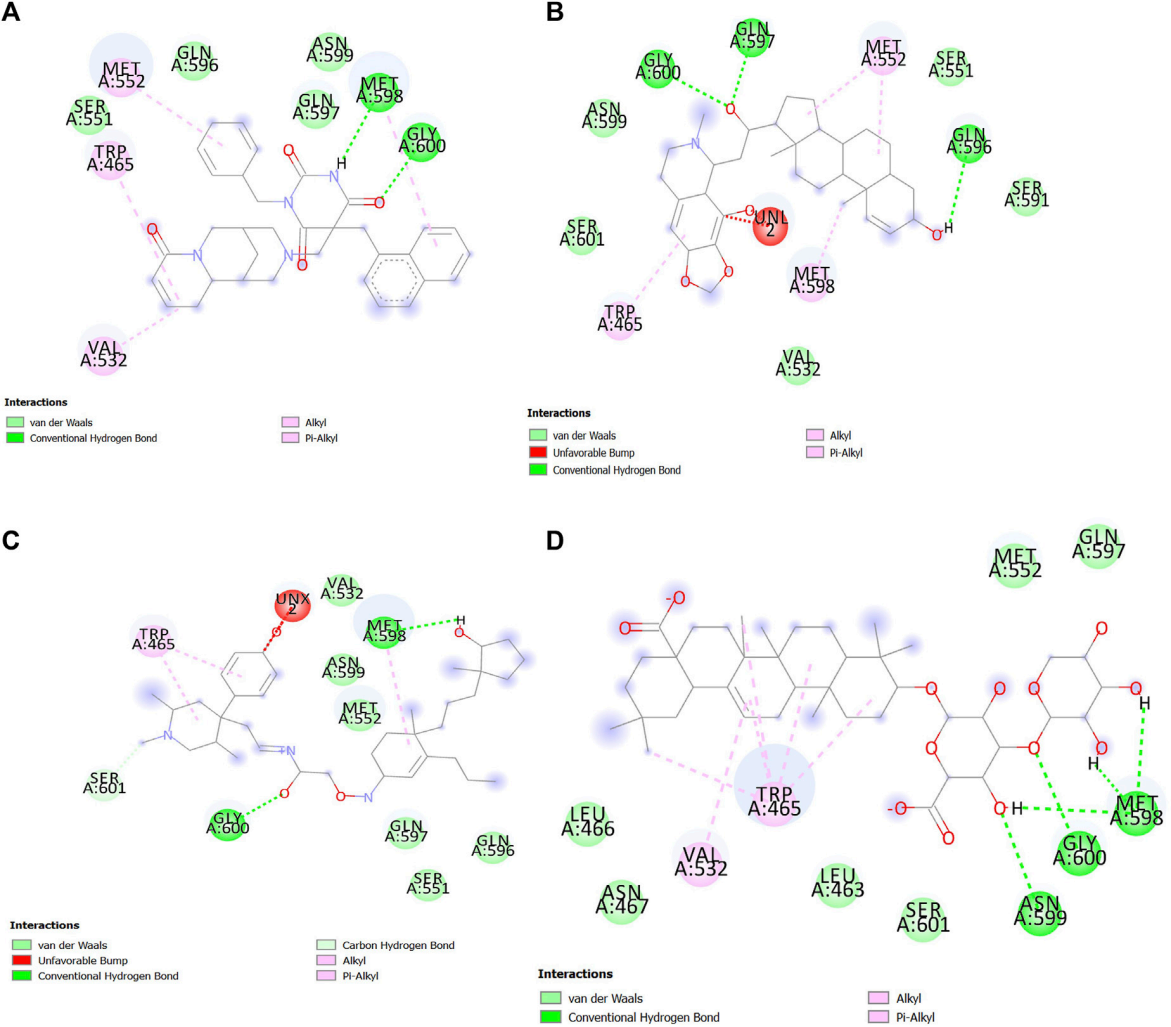
Interactions between **(A)** ZINC79204151, **(B)** ZINC33916875, **(C)** ZINC85902334, and **(D)** momordin individually with SENP1 (2D structures) in molecular docking by Discovery Studio.

Interactions between SENP1 protein and compounds ZINC79204151, ZINC85902334, and ZINC33916875 with Discovery Studio are plotted, as shown in Figure 8. In molecular docking, ZINC33916875 and momordin were found to form more hydrogen bonds, while ZINC85902334 formed more van der Waals bonds.

#### 3.6.2 Toxicity of compounds of virtual screening

The toxicity of all 20 compounds was measured by ADMET and TOPCAT criteria, as shown in Supplementary Tables S3, S4. Of these, compounds ZINC79204151, ZINC85902334, and ZINC33916875 had lower toxicity and higher docking energy. Therefore, we used them to study molecular dynamics.

AMES carcinogenicity was safe for these three compounds, and they were neither a carcinogen nor a mutagen. They can cross the blood–brain barrier and the intestinal wall except ZINC79204151 that cannot cross the blood–brain barrier. Only ZINC33916875 was permeable to CaCO_2_. All compounds could be localized in the mitochondria.

Other indicators that were evaluated at this stage show the ability to bind and suppress glycoproteins which are actively involved in the removal of xenobiotics from the cell. The ideal drug-like compounds are compounds that do not bind to glycoproteins and therefore do not leave the cell. In this case, all compounds were neither substrates for glycoproteins nor inhibitors. The point to be considered is that from these selected compounds, ideal compounds are neither glycoprotein substrates nor inhibitors. All compounds were substrates of P-gp. ZINC79204151 was not an inhibitor of P-gp. However, ZINC33916875 and ZINC85902334 were inhibitors of P-gp. Another indicator that has been measured was the possibility of metabolizing by cytochrome 450 and inhibiting this complex of metabolic proteins. The compound which cannot be metabolized can accumulate in the body and lead to unwanted side effects. ZINC79204151 was neither a substrate nor an inhibitor of CYP450. However, ZINC33916875 and ZINC85902334 were CYP3A4 substrates. Similarly, ZINC85902334 was an inhibitor of CYP3A4.

Fish toxicity (FHMT) was high for all compounds, *Tetrahymena pyriformis* toxicity (TPT) was high for all compounds, and honey bee toxicity (HBT) was low for ZINC85902334 and ZINC79204151. In addition, these compounds represented weak inhibition potential of the human ether-a-go-go-related gene (hERG), whose expression plays a significant role in the repolarization of the cardiac action potential (Sanguinetti et al., 1995).

The TOPCAT results showed all compounds are safe for the AMES mutagenicity test. Similarly, in all NTP carcinogenicity tests, ZINC85902334 was not carcinogenic. ZINC33916875 was only carcinogenic in the NTP Carcinogenicity Call (Male Rat) (v3.2) test, and ZINC79204151 was only carcinogenic in NTP Carcinogenicity Call (Male Rat) (v3.2) and NTP Carcinogenicity Call (Female Mouse) (v3.2) tests. ZINC79204151 was only carcinogenic in the FDA Carcinogenicity Female Rat Single *vs.* Mult (v3.1) test. ZINC85902334 was carcinogenic in FDA Carcinogenicity Male Rat Non vs. Carc (v3.1), FDA Carcinogenicity Male Mouse Single *vs.* Mult (v3.1), and FDA Carcinogenicity Female Mouse Single *vs.* Mult (v3.1) tests.

Developmental toxicity potential indicates mutagenic characteristics during development that can restrict their use in the pregnancy. All compounds were safe except ZINC85902334. The skin irritation test (v6.1) showed only ZINC79204151 can irritate the skin. Skin sensitization examination revealed three compounds do not cause skin allergies in the Sensitization NEG v SENS (v6.1) test, and only ZINC79204151 through the Skin Sensitization MLD/MOD v SEV (v6.1) test do not cause skin allergies. The ocular irritancy test also showed every three compounds can cause ocular irritation in the Ocular Irritancy SEV/MOD *vs.* MLD/NON (v5.1) test. In the Ocular Irritancy SEV *vs.* MOD (v5.1) test, ZINC85902334 can cause ocular irritation and in the Ocular Irritancy MLD *vs.* NON (v5.1) test, only ZINC33916875 can cause ocular irritation. We concluded using TOPCAT and ADMET properties, ZINC85902334 and ZINC33916875 have the lowest toxicity because these two compounds demonstrated more favorable factors, especially less carcinogenicity.

#### 3.6.3 Molecular dynamics results

**FIGURE 9:**
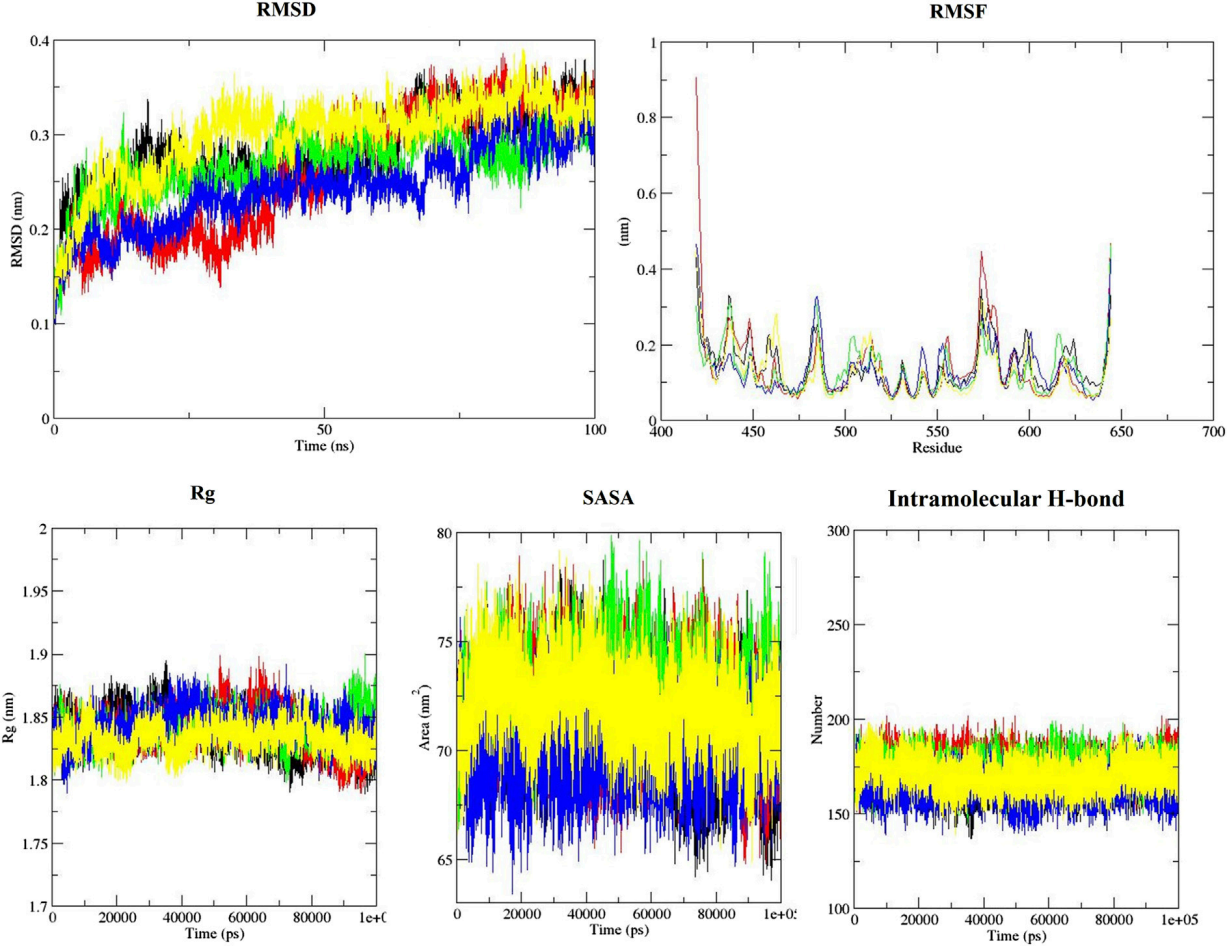
RMSD, RMSF, Rg, SASA, and intramolecular hydrogen bond plots of free-SENP1 and SENP1 complexes (black) free-SENP1, (red) ZINC79204151, (green) ZINC33916875, (blue) ZINC85902334, and (yellow) momordin.

The binding energies of ZINC79204151, ZINC85902334, and ZINC33916875 with SENP1 during molecular dynamics were also calculated with MM-PBSA to be −140.4 kcal/mol for ZINC33916875, −7.173 kcal/mol for ZINC79204151, and −84.852 kcal/mol for ZINC85902334. The RMSD was plotted to evaluate the stability of the compound complex with SENP1, as shown in Figure 9. We observed that the average RMSD is 0.26 nm for ZINC79204151, 0.24 nm for ZINC85902334, and 0.27 nm for ZINC33916875. The RMSF was also plotted to examine structural deviations in the RMSD diagram, where ZINC79204151 demonstrated the lowest fluctuations, as shown in Figure 9.

**FIGURE 10:**
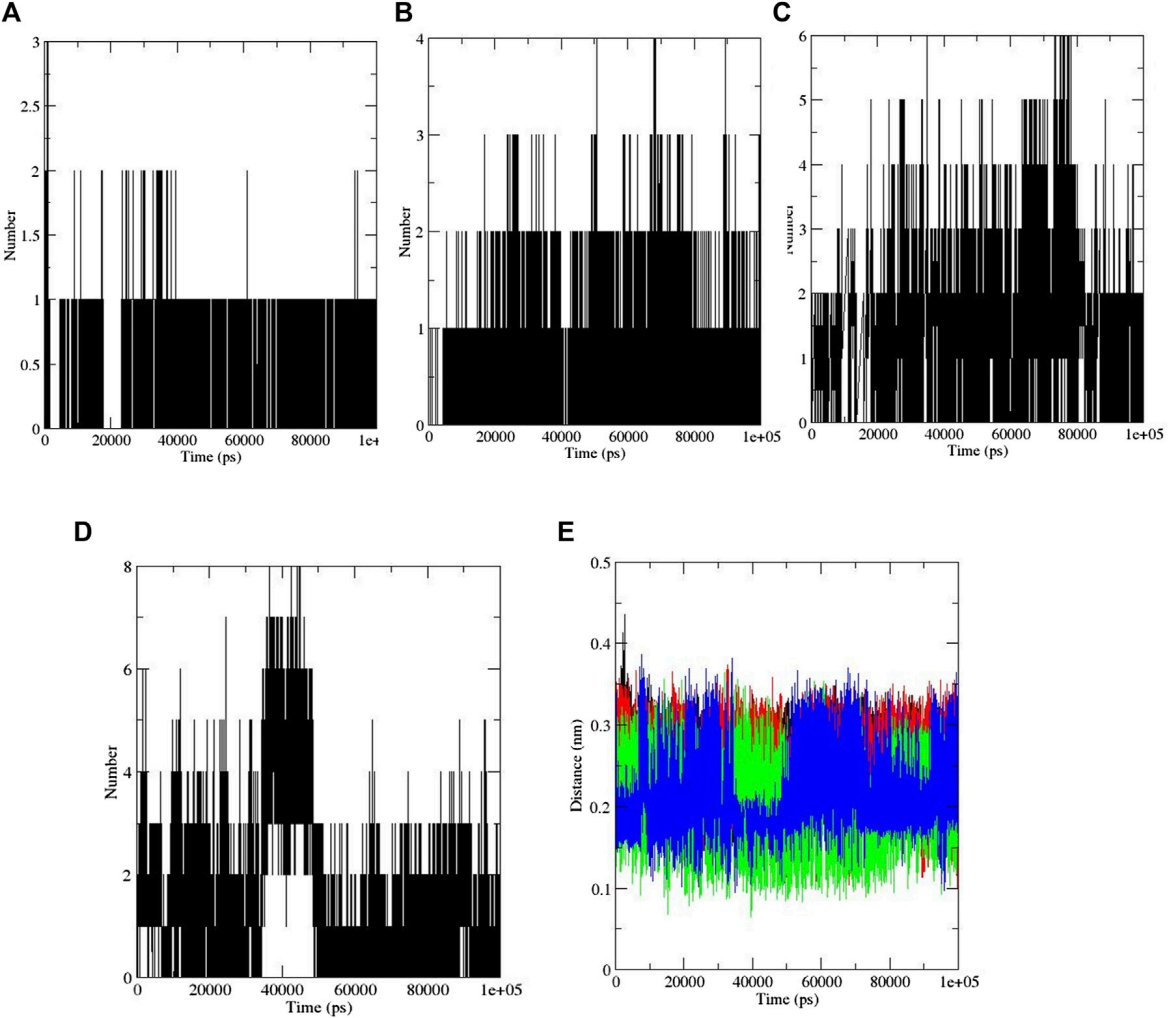
**(A)** ZINC79204151, **(B)** ZINC33916875, **(C)** ZINC85902334, and **(D)** momordin (intermolecular hydrogen bonds). **(E)** Distance plots of SENP1 complexes, (black) ZINC79204151, (red) ZINC33916875, (green) ZINC85902334, and (blue) momordin.

**FIGURE 11:**
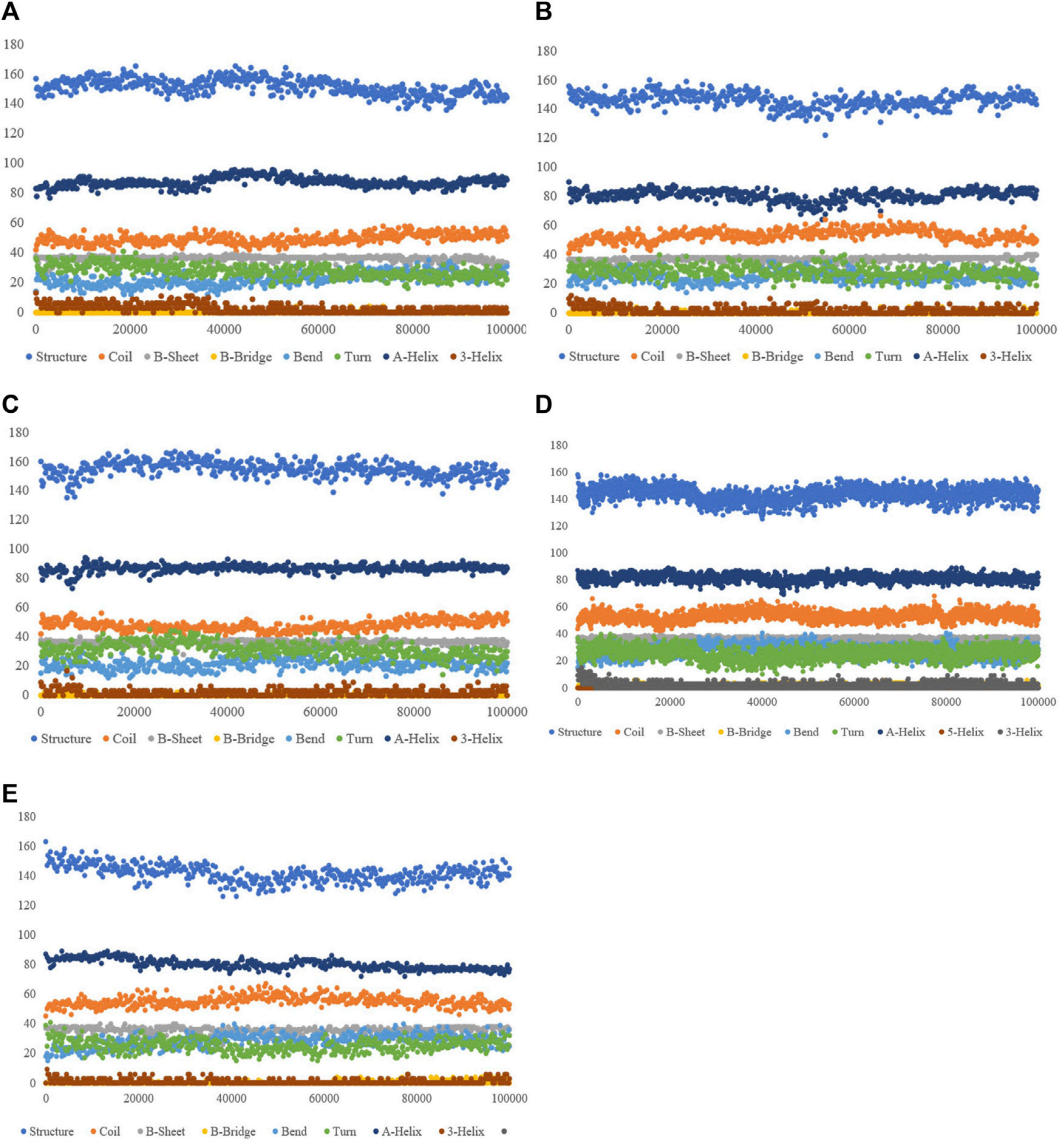
Secondary structure plots of compounds **(A)** ZINC79204151, **(B)** ZINC33916875, **(C)** ZINC85902334, **(D)** momordin, and **(E)** free-SENP1.

Study of the Rg also showed ZINC33916875 with an Rg of 1.84 nm, ZINC85902334 with an Rg of 1.84 nm, and ZINC79204151 with an Rg of 1.83 nm do not have a significant effect on protein folding, as shown in Figure 9, and SASA values for ZINC33916875, ZINC85902334, and ZINC79204151 were 73, 70, and 72 nm, respectively, which showed these compounds do not have effect on the compression and folding of the SENP1 protein, as shown in Figure 9. Intermolecular hydrogen bonds consisted of 165 for ZINC33916875, 175 for ZINC79204151, and 173 for ZINC85902334. Intermolecular hydrogen bonds were increased by two, one, and three to four hydrogen bonds for ZINC33916875, ZINC79204151, and ZINC85902334, respectively, as shown in Figures 10A, B, C. In addition, slight changes were observed in examining the secondary structure of the three compounds, as shown in Figure 11.

#### 3.6.4 Distance between SENP1 and selected compounds from virtual screening

The distance between SENP1 and ligands was obtained using the embedded packages within GROMACS. The distance between ZINC79204151, ZINC85902334, and ZINC33916875, and momordin was 0.25, 0.21, 0.26, and 0.21 nm, respectively, as shown in Figure 10E. ZINC85902334 and momordin demonstrated the lowest distance with the SENP1 protein.

#### 3.6.5 Analysis of ligand–protein interactions in molecular dynamics simulation

**FIGURE 12:**
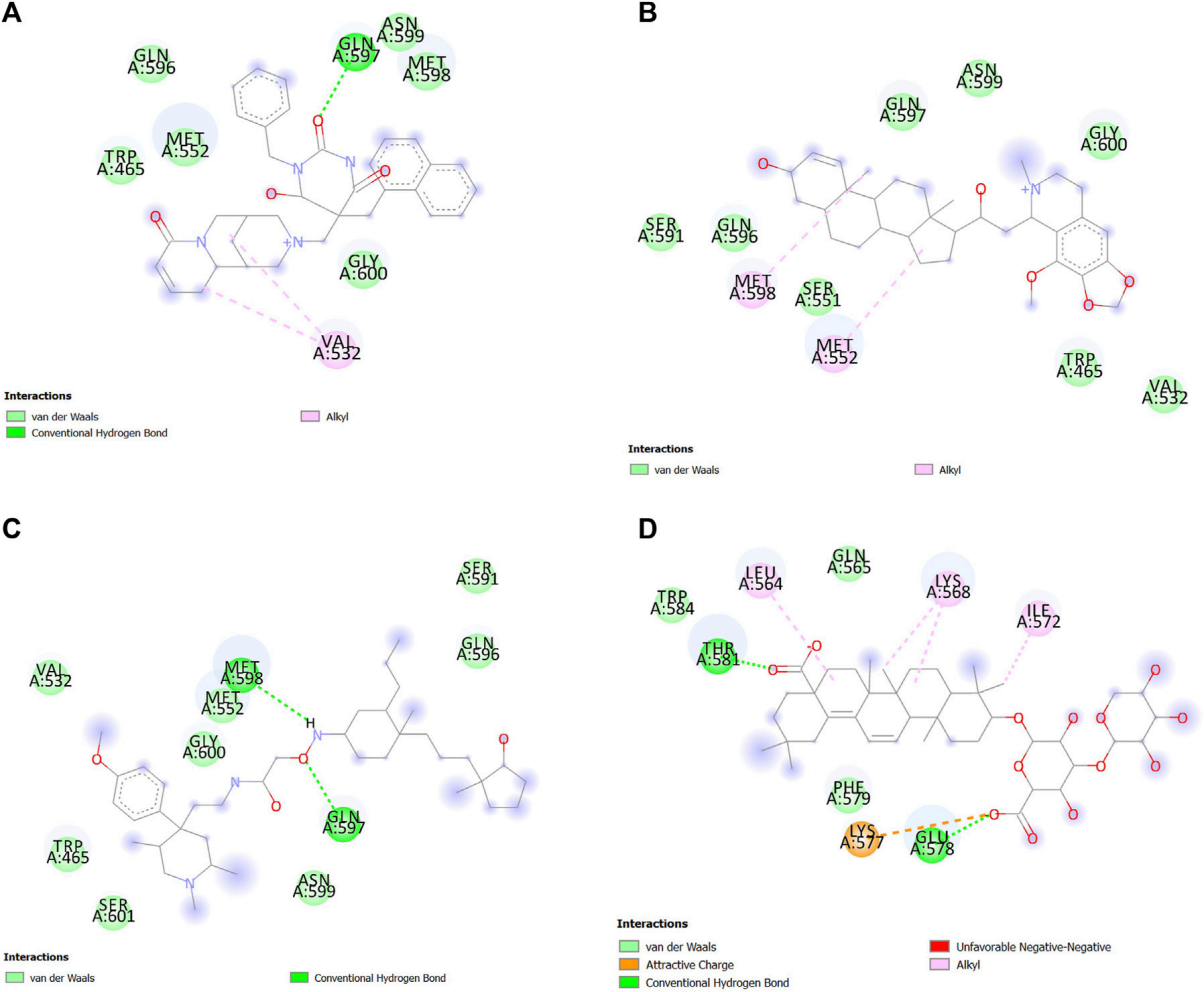
Interactions between **(A)** ZINC79204151, **(B)** ZINC33916875, **(C)** ZINC85902334, and **(D)** momordin with SENP1 by Discovery Studio after molecular dynamics simulation (2D structures).

In the molecular dynamics simulation, ZINC85902334 and momordin formed more hydrogen bonds. ZINC85902334 and ZINC33916875 bind to SENP1 with a higher number of van der Waals bonds. ZINC79204151, ZINC85902334, and ZINC33916875 bind to amino acids of the active site of SENP1, while momordin had no connection with the active site of SENP1, as shown in Figure 12.

### 3.7 Essential dynamics analysis

**FIGURE 13:**
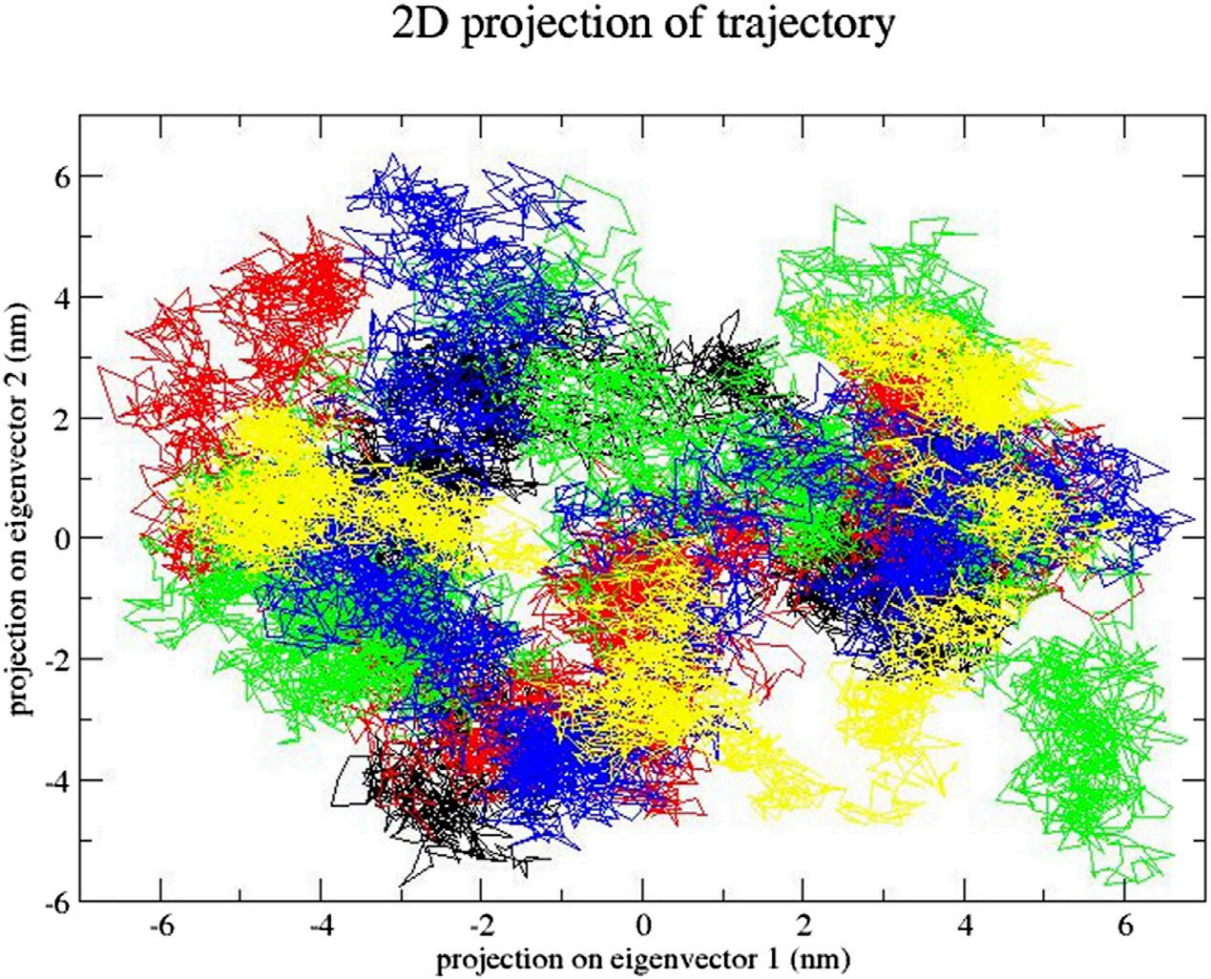
Principal component analysis. Projection of the motion for compounds (black) free-SENP1, (red) ZINC79204151, (green) ZINC33916875, and (blue) ZINC85902334.

The projection of trajectories of these compounds during the essential dynamics in the phase space along the first two principal components (PC1 and PC2) at 300 K is plotted in Figure 13. PCA showed, due to ligand binding, the structural dynamics is changing. The Figure 13 plot clearly indicates which compound–SENP1 complexes occupied less space in phase space, while the free-SENP1 occupied more space. We observed reduced flexibility of natural compound complexes than the free-SENP1.

## 4 Discussion

During the past decades, cancer has been the primary reason for death worldwide. SENP1 is a main protease in deSUMOylation. The list of SUMOylated proteins is increasing and includes proteins located in the most of the microcellular sections that are involved in cell cycle regulation, transcription, survival, and proteins involved in the cell death. The experimental results have shown the SENP1 protein is involved in cancer (Taghvaei et al., 2021b). Qiao et al. (2011) introduced benzodiazepine-based SUMO-specific protease 1 inhibitors. Chen et al. (2012) also presented 2-(4-chlorophenyl)-2-oxoethyl 4-benzamidobenzoate derivatives as a class of SENP1 inhibitors. Kumar et al. (2014) demonstrated 1,2,5-oxadiazoles as a new class of SENP1 inhibitors, and Zhao et al. (2016) identified 11 SENP1 inhibitors with various scaffolds through *in silico* screening. We also represented gallic acid can potentially inhibit SENP1 for cancer treatment (Taghvaei et al., 2021b), bethanidine can inhibit SENP1 for cardiovascular disease treatment (Taghvaei et al., 2021c), and betanin can prevent SENP2 in heart failure (Taghvaei et al., 2022). This study is the comprehensive research that includes more active compounds with stronger inhibitory properties and virtual screening of the ZINC database.

In this study, first, we examined 28 compounds for SENP1 inhibition. Among them, aspirin, berberine, cinnamic acid, ferulic acid, and resveratrol showed high docking binding energy and lowest toxicity, and then, they were selected for MDS. The MDS results demonstrated tesveratrol constitute a stable complex with SENP1, high free energy, more hydrogen bonds, more stable RMSD plot, stable Rg and SASA plots, and lower distance with SENP1. Resveratrol is a popular polyphenolic molecule that is present in various foods, such as fruits, vegetables, and chocolate. In addition, resveratrol is a popular part of grapes and wines (Poulsen et al., 2015). It has been reported that resveratrol has cytotoxic effects against several tumor cells such as myeloid, lymphoid, breast, colon, cervix, skin, stomach, ovary, prostate, liver, and thyroid carcinoma cells. Resveratrol demonstrated synergistic effect in combination with anticancer drugs in various types of cancer (Almatroodi et al., 2022). Resveratrol induces COX-2 SUMOylation likely due to its potential function as an SENP1 inhibitor (Cheng et al., 2018).

In the following section, because the compounds from the first section were not sufficient for conclusion, we studied 84,000 library of the ZINC database through molecular docking by LibDock and CDOCKER modules. Then, 20 selected compounds docked with AutoDock.4 and, at the end, compounds with higher docking binding energy and the lowest toxicity were applied to MDS. MDS of the final three compounds showed ZINC85902334 is more stable than the other two compounds, with high hydrogen bonds, strong interactions with SENP1, low RMSD, high binding energy, and lower distance with SENP1 (Kim et al., 2012; Chen et al., 2021). Therefore, ZINC85902334 binds to SENP1 powerfully.

According to our results and other results, we hope these compounds, especially resveratrol and ZINC85902334, can effectively treat various and complex diseases, especially cancer, and these compounds can be examined to treat other diseases with the overexpression of SENP1. We screened a library of the ZINC database and measured a big scale of natural compounds for SENP1 inhibition. In addition, in the best scenario, as a result of further investigations, side effects of the compounds can be reduced and the cost of anticancer drugs can be reduced. These compounds could be new leads for treatment of diverse cancer cell lines. To choose molecules with therapeutic potential, computational tools such as ADME/Tox properties, ligand-based virtual screening, and molecular dynamics (Poulsen et al., 2015) have the great importance in pharmaceutical research and industries. It is expected that the SENP1-inhibiting properties of the compounds identified in this study will be validated in wet laboratory studies for their potential as anticancer drugs.

## Data Availability

The original contributions presented in the study are included in the article/Supplementary Material. Further inquiries can be directed to the corresponding authors.
