# Supplementary material for: Computational study of SENP1 in cancer by novel natural compounds and ZINC database screening
Source: Front Pharmacol. 2023 Jul 12;14:1144632. doi: 10.3389/fphar.2023.1144632 (PMC10368881; doi:10.3389/fphar.2023.1144632)
Supplement: Supplementary file 1 [file DataSheet1.PDF]

Supplementary Material

Computational study of SENP1 in cancer by new natural compounds and ZINC database screening

Somayye Taghvaei[*a], Alireza Taghvaei[b], Mohammad Saberi[c], Chun Guo[*d], Farzaneh Sabouni[*a], Zarrin Minuchehr[*c]

a Department of Medical Biotechnology, National Institute of Genetic Engineering and Biotechnology, Tehran, Iran,

somayyetaghvaei@gmail.com, sabouni@nigeb.ac.ir

b Faculty of Pharmacy, Hamedan University of Medical Sciences, Hamedan, Iran

c Department of Systems Biotechnology, National Institute of Genetic Engineering and Biotechnology, Tehran, Iran,

minuchehr@nigeb.ac.ir

d School of Biosciences, University of Sheffield, Sheffield, S10 2TN, U.K, c.guo@sheffield.ac.uk.

*Correspondence
Farzaneh Sabouni, email: sabouni@nigeb.ac.ir
Zarrin Minuchehr, email: minuchehr@nigeb.ac.ir
Somayye Taghvaei, email: somayyetaghvaei@gmail.com

Current postal address: National Institute of Genetic Engineering and Biotechnology, Pajoohesh Blvd, Pajoohesh, km 15 Tehran – Karaj Highway, P.O. Box: 14965/161, Tehran, Iran

Chun Guo, email: c.guo@sheffield.ac.uk
Current address: School of Biosciences, University of Sheffield, Sheffield, Firth Court, Western Bank, S10 2TN, U.K

The supplementary material contains 4 Tables. Supplementary Table 1 is the ADMET results of five selected compounds of suggested compounds in the first section. Supplementary Table 2 is the TOPCAT results of five selected compounds. Supplementary Tables 3 is ADMET results of final ZINC screening. Supplementary Table 4 is TOPCAT results of the final compounds of ZINC screening.

Supplementary Table 1: ADMET results: the results of the software are using ADMETSAR (compounds: Berberine, Ferulic acid, Resveratrol, Cinnamic acid, and Aspirin.

Drug		Berberine		Ferulic acid		Resveratrol		Cinnamic acid		Aspirin	
		Results	Probability	Results	Probability	Results	Probability	Results	Probability	Results	Probability
Absorption	Blood Brain Barrier	BBB^{+}	0.9279	BBB^{-}	0.5305	BBB^{+}	0.5900	BBB^{+}	0.9526	BBB^{+}	0.9376
	Human Intestinal Absorption	HIA^{-}	0.5000	HIA^{+}	0.9614	HIA^{+}	0.9952	HIA^{+}	0.9945	HIA^{+}	0.9645
	Caco-2 Permeability	Caco^{2+}	0.8726	Caco^{2+}	0.7183	Caco^{2+}	0.8915	Caco^{2+}	0.9097	Caco^{2-}	0.6607
	P-glycoprotein S.	NS	0.6002	NS	0.5662	NS	0.6501	NS	0.8286	NS	0.6850
	P-glycoprotein I.	NI	0.8782	NI	0.9018	NI	0.9266	NI	0.9861	NI	0.9118
		NI	0.8435	NI	0.8895	NI	0.9612	NI	0.9906	NI	0.9615
	Renal Organic Cation Transporter	I	0.6035	NI	0.9086	NI	0.8634	NI	0.9145	NI	0.9140
	Subcellular Location	Mitochondria	0.4894	Mitochondria	0.83333	Mitochondria	0.6615	Plasma membrane	0.5803	Mitochondria	0.9369
Distribution	CYP450 2C9 S	NS	0.8760	NS	0.7464	NS	0.7519	NS	0.7768	NS	0.7518
Metabolism	CYP450 2D6 S.	NS	0.5937	NS	0.8922	NS	0.9288	NS	0.9644	NS	0.9116
	CYP450 3A4 S.	S	0.6738	NS	0.6289	NS	0.7143	NS	0.8188	NS	0.7225
	CYP450 1A2 I.	I	0.9107	NI	0.7513	I	0.9106	NI	0.8383	NI	0.9046
	CYP450 2C9 I.	NI	0.9070	NI	0.5793	I	0.7068	NI	0.9763	NI	0.9071
	CYP450 2D6 I.	I	0.8933	NI	0.9588	NI	0.9226	NI	0.9546	NI	0.9576
	CYP450 2C19 I.	NI	0.7463	NI	0.6276	I	0.8052	NI	0.9724	NI	0.9446
	CYP450 3A4 I.	NI	0.5873	NI	0.9240	I	0.7539	NI	0.9702	NI	0.9611
	CYP Inhibitory Promiscuity	High	0.9003	Low	0.7745	High	0.8559	High	0.9687	High	0.9557
Excretion	Human Ether-a-	Weak Inhibitor	0.8367	Weak Inhibitor	0.9754	Weak Inhibitor	0.8933	Weak Inhibitor	0.9620	Weak Inhibitor	0.9433
Toxicity	go-go-Related Gene inhibition	NI	0.8734	NI	0.9575	NI	0.9462	NI	0.9899	NI	0.9799
	AMES Toxicity	Non- AMES toxic	0.9132	Non- AMES toxic	0.9132	Non- AMES toxic	0.8407	Non- AMES toxic	0.9722	Non- AMES toxic	0.9326
	Carcinogens	Non-carcinogens	0.9539	Non-carcinogens	0.9076	Non-carcinogens	0.7825	Non-carcinogens	0.5927	Non-carcinogens	0.8356
	Fish Toxicity (FHMT)	Low	0.5187	High	0.9325	High	0.8824	High	0.8969	High	0.9391
	Tetrahymena Pyriformis Toxicity (TPT)	High	0.7251	High	0.9727	High	0.7771	High	0.9585	High	0.8519
	Honey Bee Toxicity (HBT)	Low	0.5868	High	0.7640	High	0.8051	High	0.7685	High	0.7453
	Biodegradation	Not ready biodegradable	0.8408	Ready biodegradable	0.7554	Not ready biodegradable	0.8499	Ready biodegradable	0.7942	Ready biodegradable	0.9067
	Acute Oral Toxicity	III	0.6360	IV	0.6265	III	0.6825	III	0.8487	II	0.7260
	Carcinogenicity (three-class)	Non-R	0.4717	Non-R	0.5903	Non-R	0.5753	Non-R	0.7458	Non-R	0.7139
Absorption	Aqueous solubility, LogS	-2.9737	LopS	-2.4766	LopS	-2.7776	LopS	-2.4174	LopS	-1.7826	LopS
	Caco-2 Permeability, LogPapp,	1.2267	LogPapp, cm/s	0.6802	LogPapp, cm/s	1.1325	LogPapp, cm/s	1.8973	LogPapp, cm/s	0.5054	LogPapp, cm/s

	cm/s										
Toxicity	Rat Acute Toxicity LD50, mol/kg	2.7834	LD50, mol/kg	1.4314	LD50, mol/kg	1.6791	LD50, mol/kg	1.7416	LD50, mol/kg	2.6386	LD50, mol/kg
	Fish Toxicity pLC50 mg/L	1.0011	pLC50.	1.2433	pLC50.	0.6165	pLC50.	1.6417	pLC50.	-0.1352	pLC50.
	Tetrahymena Pyriformis Toxicity pIGC50, ug/L	0.2988	pIGC50,	1.0404	pIGC50,	0.6410	pIGC50,	0.1061	pIGC50,	0.2236	pIGC50,

Supplementary Table 2: TOPKAT results for compounds, results of the TOPKAT module in the Discovery Studio 2.5, (Compounds: Berberine, Ferulic acid, Resveratrol, Cinnamic acid, and Aspirin**.**

	**Berberin**	**Ferulic acid**	**Resveratrol**	**Cinnamic acid**	**Aspirin**
Ames Mutagenicity (v3.1)	0.000	0.015	0.000	0.964	0.000
NTP Carcinogenicity Call (Male Rat) (v3.2)	1.000	0.034	0.000	0.021	0.967
NTP Carcinogenicity Call (Female Rat) (v3.2)	1.000	0.001	0.002	0.998	0.000
NTP Carcinogenicity Call (Male Mouse) (v3.2)	1.000	0.000	0.000	0.236	0.000
NTP Carcinogenicity Call (Female Mouse) (v3.2)	1.000	0.989	0.051	0.002	0.005
FDA Carcinogenicity Male Rat Non vs Carc (v3.1)	0.017	1.000	1.000	0.603	1.000
FDA Carcinogenicity Male Rat Single vs Mult (v3.1)	1.000	0.885	0.000	0.988	0.000
FDA Carcinogenicity Female Rat Non vs Carc (v3.1)	1.000	0.001	0.000	0.090	0.400
FDA Carcinogenicity Female Rat Single vs Mult (v3.1)	1.000	0.000	0.000	0.109	0.000
FDA Carcinogenicity Male Mouse Non vs Carc (v3.1)	1.000	0.001	0.000	0.120	0.024
FDA Carcinogenicity Male Mouse Single vs Mult (v3.1)	1.000	0.000	0.274	0.947	0.000
FDA Carcinogenicity Female Mouse Non vs Carc (v3.1)	0.001	0.000	0.000	1.000	0.256
FDA Carcinogenicity Female Mouse Single vs Mult (v3.1)	0.000	1.000	0.993	0.019	0.999
Weight of Evidence Carcinogenicity Call (v5.1)	1.000	0.000	1.000	0.005	0.000
Developmental Toxicity Potential (DTP) (v3.1)	0.721	0.000	0.000	0.010	0.000
Rat Oral LD50 (v3.1) (mg/kg)	371.3	10	10	1.5	6.3
Rat Maximum Tolerated Dose - Feed/Water (v6.1) (mg/kg)	13.2	846.6	336.3	2.6	67.8
Rat Maximum Tolerated Dose - Gavage (v6.1) (mg/kg)	36.5	2.3	929.4	2.6	24.9
Rat Inhalational LC50 (v6.1) (mg/m3/H)	451.3	10	3.4	10	10
Chronic LOAEL (v3.1) (mg/kg)	31.6	809	65.2	84.0	30.1

Skin Irritation (v6.1)	1.000	0.000	0.503	0.011	0.000
Skin Sensitization NEG v SENS (v6.1)	0.000	0.994	0.970	0.142	1.000
Skin Sensitization MLD/MOD v SEV (v6.1)	0.000	0.000	0.000	0.000	0.000
Ocular Irritancy SEV/MOD vs MLD/NON (v5.1)	0.072	0.134	0.996	0.654	0.992
Ocular Irritancy SEV vs MOD (v5.1)	1.000	1.000	0.084	0.139	0.899
Ocular Irritancy MLD vs NON (v5.1	0.000	1.000	0.089	0.408	1.000
Aerobic Biodegradability (v6.1)	1.000	1.000	0.000	1.000	0.000
Fathead Minnow LC50 (v3.2) (mg/l)	45.7	2.8	78.6	930.2	1.4
Daphnia EC50 (v3.1) (mg/l)	64.1	19.6	1000	12.2	5.0
LogP (v3.1)	4.168	-0.220	0.731	2.200	0.829

*0.0 to 1.0: Probable calculated values for each of the compounds, 0.0 to 0.3: Negative response of the compounds in the laboratory tests, 0.7 to 1.0: Positive response in these experiments, 0.3 to 0.7: An intermediate state, and greater than 1.0: Amounts were consumed by these compounds

Supplementary Table 3: ADMET results: The results of the software are using ADMETSAR (compounds: ZINC79204151, ZINC85902334, and ZINC33916875

		ZINC79204151		ZINC85902334		ZINC33916875	
Drug		Results	Probability	Results	Probability	Results	Probability
Absorption	Blood Brain Barrier	BBB^{-}	0.7193	BBB^{+}	0.5687	BBB^{+}	0.9547
	Human Intestinal	HIA^{+}	0.8272	HIA^{+}	0.9946	HIA^{+}	0.9566
	Absorption						
	Caco-2 Permeability	Caco^{2-}	0.5644	Caco^{2-}	0.6458	Caco^{2-}	0.6210
	P-glycoprotein S.	S	0.6932	S	0.9174	S	0.8837
	P-glycoprotein I.	NI	0.8015	I	0.7882	NI	0.8522
		NI	0.6469	I	0.9571	NI	0.6959
	Renal	NI	0.7518	NI	0.7204	NI	0.5414
	Organic Cation						
	Transporter						
	Subcellular Location	Mitochondria	0.9083	Mitochondria	0.5537	lysosome	0.5509
Distribution	CYP450 2C9 S	NS	0.8053	NS	0.8085	NS	0.9041
Metabolism	CYP450 2D6 S.	NS	0.7093	NS	0.7829	NS	0.6046
	CYP450 3A4 S.	NS	0.5000	S	0.7975	S	0.7856
	CYP450 1A2 I.	NI	0.5458	NI	0.8856	NI	0.9294
	CYP450 2C9 I.	NI	0.5272	NI	0.7629	NI	0.9289
	CYP450 2D6 I.	NI	0.6905	NI	0.7773	NI	0.7274
	CYP450 2C19 I.	NI	0.7236	NI	0.7324	NI	0.8962
	CYP450 3A4 I.	NI	0.5689	I	0.5531	I	0.6384
	CYP Inhibitory	Low	0.5354	Low	0.8748	High	0.9359
	Promiscuity						
Excretion	Human Ether-a-	Weak	0.9588	Weak	0.9559	Weak	0.7407
Toxicity	go-go-Related Gene	Inhibitor		Inhibitor		Inhibitor	
	inhibition	I	0.5144	I	0.6286	NI	0.8337
	AMES Toxicity	Non- AMES	0.7989	Non- AMES	0.6197	Non- AMES	0.6482
		toxic		toxic		toxic	
	Carcinogens	Non- carcinogens	0.8308	Non- carcinogens	0.8087	Non- carcinogens	0.9254
	Fish Toxicity	High	0.9075	High	0.9810	High	0.9892
	(FHMT)						
	Tetrahymena	High	0.9715	High	0.9632	High	0.9950
	Pyriformis Toxicity						
	(TPT)						
	Honey Bee Toxicity	Low	0.6190	Low	0.5950	High	0.6053
	(HBT)						
	Biodegradation	Not ready	1.0000	Not ready	0.9883	Not ready	0.9194
		biodegradable		biodegradable		biodegradable	
	Acute Oral Toxicity	III	0.6043	III	0.5795	III	0.5519
	Carcinogenicity	Non-R	0.6113	Non-R	0.5367	Non-R	0.4382
	(three-class)						

Absorption	Aqueous solubility, LogS	-3.3465	LopS	-3.0585	LopS	-2.7797	LopS
	Caco-2 Permeability, LogPapp, cm/s	0.5534	LogPapp, cm/s	0.3702	LogPapp, cm/s	0.6 073	LogPapp, cm/s
Toxicity	Rat Acute Toxicity LD50, mol/kg	2.3097	LD50, mol/kg	2.8440	LD50, mol/kg	2.4440	LD50, mol/kg
	Fish Toxicity pLC50 mg/L	1.0609	pLC50.	1.1728	pLC50.	1.1353	pLC50.
	Tetrahymena Pyriformis Toxicity pIGC50, ug/L	0.9572	pIGC50,	0.5839	pIGC50,	0.3500	pIGC50,

Supplementary Table 4: TOPKAT results for compounds, results of the TOPKAT module in the Discovery Studio 2.5, (Compounds: ZINC33916875, ZINC79204151, and ZINC85902334.

	**ZINC33916875**	**ZINC79204151**	**ZINC85902334**
Ames Mutagenicity (v3.1)	0.000	0.000	0.000
NTP Carcinogenicity Call (Male Rat) (v3.2)	0.990	1.000	0.000
NTP Carcinogenicity Call (Female Rat) (v3.2)	0.001	0.000	0.006
NTP Carcinogenicity Call (Male Mouse) (v3.2)	0.000	0.000	0.000
NTP Carcinogenicity Call (Female Mouse) (v3.2)	0.001	1.000	0.000
FDA Carcinogenicity Male Rat Non vs Carc (v3.1)	1.000	0.000	1.000
FDA Carcinogenicity Male Rat Single vs Mult (v3.1)	0.000	0.537	0.000
FDA Carcinogenicity Female Rat Non vs Carc (v3.1)	1.000	0.001	0.003
FDA Carcinogenicity Female Rat Single vs Mult (v3.1)	0.000	1.000	0.000
FDA Carcinogenicity Male Mouse Non vs Carc (v3.1)	0.999	0.000	0.182
FDA Carcinogenicity Male Mouse Single vs Mult (v3.1)	1.000	0.000	1.000
FDA Carcinogenicity Female Mouse Non vs Carc (v3.1)	0.002	0.000	0.000
FDA Carcinogenicity Female Mouse Single vs Mult (v3.1)	1.000	0.000	1.000
Weight of Evidence Carcinogenicity Call (v5.1)	1.000	0.000	1.000
Developmental Toxicity Potential (DTP) (v3.1)	0.000	1.000	1.000
Rat Oral LD50 (v3.1) (mg/kg)	52.7	273.5	1.1
Rat Maximum Tolerated Dose - Feed/Water (v6.1) (mg/kg)	5.6	360.1	4.6
Rat Maximum Tolerated Dose - Gavage (v6.1) (mg/kg)	15.4	997.1	12.8

Rat Inhalational LC50 (v6.1) (mg/m3/H)	18.6	10	809.4
Chronic LOAEL (v3.1) (mg/kg)	187.9	10	660
Skin Irritation (v6.1)	1.000	0.000	1.000
Skin Sensitization NEG v SENS (v6.1)	0.848	1.000	1.000
Skin Sensitization MLD/MOD v SEV (v6.1)	0.000	1.000	0.000
Ocular Irritancy SEV/MOD vs MLD/NON (v5.1)	0.000	0.000	0.000
Ocular Irritancy SEV vs MOD (v5.1)	1.000	1.000	0.000
Ocular Irritancy MLD vs NON (v5.1	0.085	1.000	0.464
Aerobic Biodegradability (v6.1)	0.000	0.000	0.000
Fathead Minnow LC50 (v3.2) (mg/l)	185.8	174.6	546.8
Daphnia EC50 (v3.1) (mg/l)	1000	112.8	1000
LogP (v3.1)	4.172	5.042	4.270

*0.0 to 1.0: Probable calculated values for each of the compounds, 0.0 to 0.3: Negative response of the compounds in the laboratory tests, 0.7 to 1.0: Positive response in these experiments, 0.3 to 0.7: An intermediate state, and greater than 1.0: Amounts were consumed by these compounds
